# Physiological and Biochemical Responses of Two Major Legume Crops to Seed Priming—A Systematic Review

**DOI:** 10.3390/plants15111636

**Published:** 2026-05-26

**Authors:** Lungani Ngcungama, Sandiswa Figlan, Phumzile Pretty Sibisi, Maltase Mutanda, Mhlonipheni Msomi

**Affiliations:** Department of Agriculture and Animal Health, University of South Africa, Florida, Roodepoort 1709, South Africa; figlas@unisa.ac.za (S.F.); sibispp@unisa.ac.za (P.P.S.); 19283849@mylife.unisa.ac.za (M.M.); msomimn@unisa.ac.za (M.M.)

**Keywords:** seed priming, common bean, soybean, tropical climate, proline

## Abstract

Seed priming is recognized as an environmentally friendly technique to enhance the physiological and biochemical performance of crops. However, its effectiveness varies depending on factors such as crop type, priming agents and climatic conditions. Based on this hypothesis, this comparative, climate- and soil-dependent meta-synthesis study therefore aimed to evaluate how these factors shape plant responses in common bean (*Phaseolus vulgaris* L.) and soybean (*Glycine max* L.), while providing insights into sustainable strategies for improving crop performance, food security, and progress toward sustainable development goals. A cross-study synthesis of 31 peer-reviewed articles from Web of Science, Scopus, and Google Scholar evaluated the influence of these factors on key physiological traits, chlorophyll content (CC) and net photosynthesis rate (Pn), and biochemical traits, proline (Pro), superoxide dismutase (SOD), and catalase (CAT) activity. The findings indicated greater priming-induced enhancements in common bean than soybean for most traits: chlorophyll content (36.6% in common bean and 25.6% in soybean), net photosynthesis rate (33.2% in common bean and 19.8% in soybean), proline content (45.2% in common bean and 40.9% in soybean), and SOD activity (37.1% in common bean and 30.5% in soybean). Soybean only showed superior enhancement in CAT activity (40.1% in soybean and 19.5% in common bean). The climatic conditions impacted the outcomes, with physiological traits (CC and Pn) responding more prominently under semi-arid and arid climates, and biochemical traits (Pro, CAT and SOD) showing higher responsiveness in continental and Mediterranean climates. Significant (*p* < 0.05) correlations were found between CAT activity and priming agents (r = 0.54); SOD and crop type (r = 0.52); and Pn and crop type (0.90). Multivariate analysis revealed that soybean was positively associated with silt, clay, pH, tropical climate and CAT activity, while common bean was linked to nitrogen, arid conditions, SOD activity and proline. These differences could be due to the molecular and genetic variations in the two crops. Unlike previous reviews, this study provides the first quantitative synthesis integrating crop type, priming agents, and climatic variables, aiming to evaluate how these factors influence the responses of two major legume crops to seed priming. Overall, the findings highlight the need for crop- and environment-specific priming protocols to optimize the benefits of seed priming as a cost-effective approach to enhance crop performance and productivity.

## 1. Introduction

Leguminous crops play a critical role in global food security by contributing significantly to both human nutrition and agricultural productivity [1,2]. Among these, soybean (*Glycine max* L.) and common bean (*Phaseolus vulgaris* L.) stand out as major species with wide-ranging economic and nutritional importance [3,4]. These legumes are a rich source of high-quality proteins, starch, dietary fibers, vitamins, folic acid, and essential minerals, making them key dietary components worldwide [5,6]. Additionally, they contribute to soil health through biological nitrogen fixation via symbiosis with bacteria like *Rhizobium leguminosarum bv. phaseoli*, reducing the need for nitrogen fertilizers [7,8]. Despite these significances, soybean and common bean are adversely affected by both biotic (e.g., pests and diseases) and abiotic stresses (e.g., drought stress and soil salinity). These stressors cause modifications in the physiological and biochemical traits of soybean and common bean [9,10], which affect their productivity and compromise the yield nutritional quality. The global demand for these crops has been growing at a compound annual growth rate estimated at over 6% over the past decade, largely driven by a preference for plant-based protein over animal-based sources [11]. This increased demand places significant pressure on farmers to maintain productivity while facing escalating environmental challenges [12].

Abiotic and biotic stressors adversely affect physiological traits including transpiration rates, stomatal conductance, net photosynthesis, and chlorophyll content in plants [13,14]. These stressors cause biochemical changes, including promoting the accumulation of reactive oxygen species, e.g., superoxide radical (O_2_^•−^), hydroxyl radical (•OH) and hydrogen peroxide (H_2_O_2_), leading to oxidative damage [15]. In addition, oxidative stress reduces key biochemical compounds such as sucrose, starch, and soluble proteins in the leaves and seeds [16]. Therefore, it is crucial to develop strategies that improve the resilience of these plants against both biotic and abiotic stressors [17,18].

Various researchers have proposed mitigation strategies, including cultural practices [19,20], breeding of stress resistant or tolerant cultivars [21,22], pesticide application [23,24] and seed priming [25,26]. Cultural methods such as weeding, crop rotation, and mulching are effective but labor intensive and time consuming [27,28]. Breeding for resistant cultivars can present challenges of linkage drag and trade-offs, and high costs associated with sophisticated equipment, as well as analysis, pathogen evolution and resistance breakdown [29,30]. Breeding programs that improve legume crops also depend largely on multi-environmental field trials and genetic studies to identify the best-performing genotypes across diverse geographical regions [31,32]. On the other hand, the use of chemical pesticides has detrimental effects on the environment and risks to human health [33,34] and some of the pests might evolve and become resistant to pesticides [35,36]. Among these aforementioned control strategies, seed priming stands out as a simple, cost-effective, and environmentally friendly approach that enhances plant performance by triggering beneficial physiological and biochemical changes before seed germination [37,38]. Seed priming involves controlled hydration or biochemical pretreatment that activates metabolic and stress-protective processes within the seed, promoting faster, more uniform germination and greater tolerance to environmental stresses [39]. Unlike other seed treatments, such as seed coating or seed dressing, which primarily provide external protection or nutrients, seed priming preconditions the seed’s internal machinery [40]. It induces a form of defense memory, allowing for a faster and more robust response to subsequent biotic and abiotic stresses. This memory is mediated through complex signaling networks, including the salicylic acid (SA) and jasmonic acid (JA) pathways, reactive oxygen species (ROS) signaling, epigenetic regulation, and reprogramming of stress-related genes involved in antioxidant defense and metabolic adjustments [41]. Priming techniques include soaking seeds in water (hydropriming), using low-water potential solutions like polyethylene glycol or salt solutions (osmo-priming), applying plant growth regulators, polyamines (hormonal priming), plant growth-promoting bacteria (biopriming), macro- or micronutrients (nutri-priming), or natural plant extracts [42].

Research has shown significant different physiological and biochemical responses between primed and non-primed common bean and soybean seeds. For common bean, Ahmed et al. [26] reported that priming seeds with 20 µmol L^−1^ triacontanol significantly enhanced stomatal conductance, photosynthetic rate, and transpiration rate by 24%, 52%, and 71%, respectively, compared to unprimed plants. Concurrently, Borromeo et al. [43] observed that priming common bean seeds with 40 mM sodium chloride under saline stress resulted in a significant increase in phenolic content, proline levels, and enzyme activities (SOD and CAT), with increases of 50%, 43%, 63%, and 105.75%, respectively. Studies on soybean seeds have also demonstrated beneficial effects of priming treatments on physiological and biochemical traits. Baghel et al. [44] found that priming soybean seeds with a static magnetic field at 100 mT for 1 h led to significant increases in chlorophyll a, chlorophyll b, photosynthetic rate, stomatal conductance, and transpiration. Furthermore, Jaybhaye et al. [45] reported that priming soybean seeds with gibberellic acid resulted in an increase in proline levels, SOD, and CAT by 81%, 31%, and 62%, respectively, compared to the unprimed plants.

However, not all priming treatments consistently yield positive results. Variability in climatic conditions, crop types, soil properties, priming duration and priming agents can influence the outcomes, with even some treatments showing limited and negative effects [46,47]. These factors play a critical role, with some studies reporting that pronounced benefits may only be under specific conditions [48]. Differences in climatic conditions, crops and priming agents influence the physiological and biochemical responses of soybean and common bean to seed priming. Therefore, this study aimed to evaluate the extent to which these factors shape the plants’ responses and to provide insights into sustainable strategies for improving the crops’ performances, food security, and progress toward sustainable development goals.

## 2. Methodology

### 2.1. Study Setup

**Figure 1:**
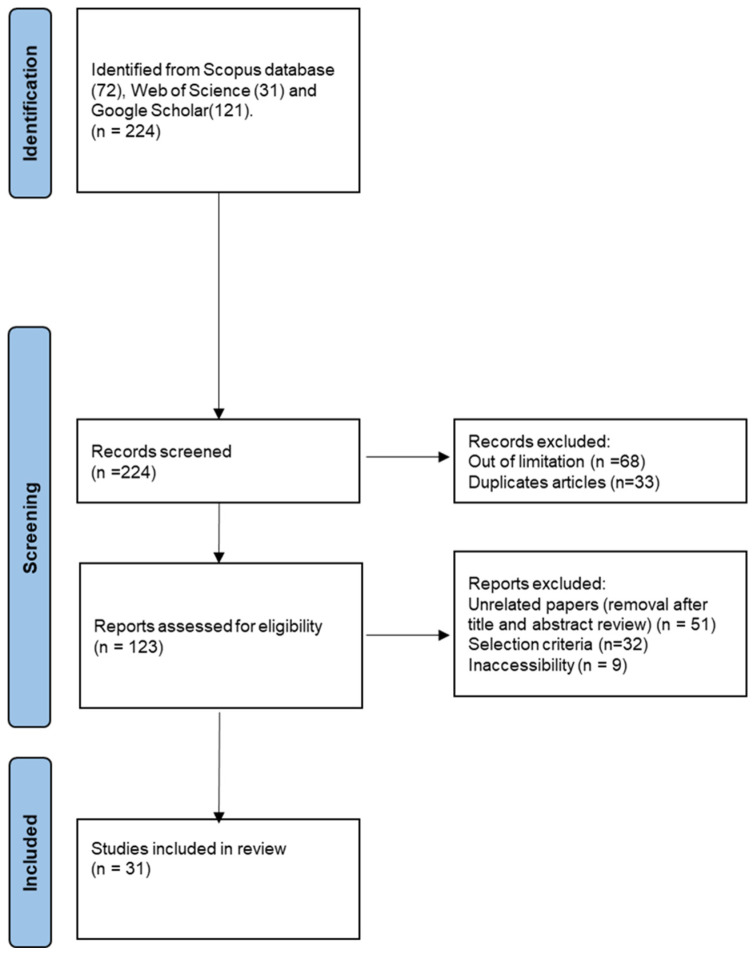
Prisma flow diagram for the current study.

The systematic literature review was conducted in accordance with the Preferred Reporting Items for Systematic Reviews and Meta-Analysis for Scoping Reviews (PRISMAScR) guidelines [49]. Research articles published between 2010 and 2024 and reporting on seed priming on physiological and biochemical variables for soybean and common bean were identified using Google Scholar, Scopus, and Web of Science. The keywords used to identify relevant articles were “seed priming”, “legume crops”, “soybean”, “common bean”, “physiological traits”, and “biochemical traits”. All relevant articles were entered into a Microsoft Excel database. Articles included in the database had to meet the following criteria: (i) they needed to report on either physiological or biochemical traits, (ii) they had to report on data for either soybean or common bean; (iii) and they needed to provide data on the studied traits for both primed and control treatments. We included studies conducted under both controlled and open-field conditions. The final database (summarized in Table 1 and Figures S1–S5) consists of 31 research articles which remained suitable after the inclusion criteria. The flow diagram for the assessment of studies identified in the systematic review is shown in Figure 1. The physiological and biochemical traits considered for the study included chlorophyll content (CC), photosynthesis (Pn), proline (Pro), superoxide dismutase (SOD), and catalase (CAT).

### 2.2. Definition of Environmental Variables

The observations in the final database were stratified using long-term climate conditions (mean annual precipitation [MAP] and mean annual temperature [MAT]) and soil properties (pH, bulk density, nitrogen, phosphorus, potassium, clay, silt, and sand contents) (Table 2). In cases where the MAP and MAT were not provided in the papers, the data was obtained from [50]. Those climatic categories were subtropical, tropical, temperate, arid, semi-arid, continental and Mediterranean. Soil bulk density (BD) was cited from the articles, and where BD was given for different horizons, the average for the whole profile was calculated. The soil pH used in the current paper is based on the calcium chloride (CaCl_2_) scale averaged across the soil profile. A summary of the classification for all environmental factors and soil properties used is provided in Table 3.

**Table 1:** References included in the database with location, crop names, priming agents, environmental factors (MAT and MAP) and data considered from the study.

No	Author	Crop Name	Priming Agent	Climate	Country	Continent	MAP (mm yr^−1^)	MAT (°C yr^−1^)	Data Considered for the Study
1	Al Hijab et al. [51]	Common bean	Bio-priming	Arid	Egypt	Africa	51	27	SOD, CAT
2	Borromeo et al. [43]	Common bean	Halo-priming	Mediterranean	Italy	Europe	750	26	CC
3	Eisvand et al. [52]	Common bean	Hormonal priming	Mediterranean	Iran	Asia	463	25	CC
4	Jaybhaye et al. [45]	Soybean	Hormonal priming	Tropical	India	Asia	947	23	Pro, SOD, CAT
5	Mansoor et al. [53]	Common bean	Nutri-priming	Subtropical	India	Asia	1100	25	Chl, Pro
6	Abdulmajeed et al. [54]	Common bean	Hormonal priming	Arid	Saudi Arabia	Asia	59	22	CC, Pro
7	Ahmed et al. [26]	Common bean	Nano-priming	Semi-arid	Pakistan	Asia	630	25	CC
8	Farajollahi et al. [55]	Soybean	Nano-priming	Mediterranean	Iran	Asia	530	25	Pn, SOD, CAT
9	Mansoor et al. [56]	Common bean	Nutri-priming	Subtropical	India	Asia	1083	26	CC, Pro
10	Perfileva et al. [57]	Soybean	Nano-priming	Continental	Russia	Europe	496	23	CAT
11	Rady et al. [58]	Common bean	Bio-priming	Arid	Egypt	Africa	140	26	CC, SOD, CAT
12	Alinia et al. [59]	Common bean	Hormonal priming	Semi-arid	Iran	Asia	350	27	CC, Pn, SOD, CAT
13	Alinia et al. [60]	Common bean	Hormonal priming	Semi-arid	Iran	Asia	350	23	SOD
14	Lastochkina et al. [61]	Common bean	Bio-priming	Moderate	Russia	Europe	550	25	CC, Pro
15	Goiba et al. [62]	Soybean	Halo-priming	Semi-arid	India	Asia	580	26	CC
16	Mangena et al. [63]	Soybean	Hormonal priming	Semi-arid	South Africa	Africa	473	24	CC
17	Nazari et al. [64]	Soybean	Chemical priming	Semi-arid	Iran	Asia	169	25	SOD, CAT
18	Sheteiwy et al. [65]	Soybean	Hormonal priming	Subtropical	China	Asia	1256	28	CC, Pn, SOD, CAT
19	Soliman et al. [66]	Soybean	Hormonal priming	Arid	Egypt	Africa	41	28	CC, Pn
20	Sofy et al. [67]	Common bean	Osmo-priming	Tropical	Egypt	Africa	20	27	CC, Pro, SOD, CAT
21	Tabesh et al. [68]	Common bean	Nutri-priming	Semi-arid	Morocco	Africa	700	30	CC
22	Abdel-Aziz et al. [69]	Common bean	Nano-priming	Arid	Egypt	Africa	150	23	Pn, SOD, CAT
23	Majda et al. [70]	Common bean	Nutri-priming	Mediterranean	Morocco	Africa	500	25	CC
24	Dai et al. [71]	Soybean	Hydro-priming	Continental	China	Asia	550	25	Pro, SOD, CAT
25	Keshavarz et al. [72]	Common bean	Nutri-priming	Mediterranean climate	Iran	Asia	250	22	CC, Pro, SOD, CAT
26	Langeroodi et al. [73]	Soybean	Hydro-priming	Semi-arid	Iran	Asia	230	30	CC, Pro, SOD, CAT
27	Keshavarz et al. [74]	Common bean	Nutri-priming	Semi-arid	Iran	Asia	230	22	CC, Pro, SOD, CAT
28	Semida et al. [75]	Common bean	Bio-priming	Arid	Egypt	Africa	200	25	Pro, SOD, CAT
29	Syatrianty et al. [76]	Soybean	Osmo-priming	Tropical	Indonesia	Asia	3357	26	CC
30	Rady et al. [77]	Common bean	Bio-priming	Arid	Egypt	Africa	80	18	CC, Pro, SOD, CAT
31	Ghassemi-Golezani et al. [78]	Common bean	Hydro-priming	Semi-arid	Iran	Asia	285	21	CC

### 2.3. Definition of Considered Trait Variables

The biochemical and physiological traits used in this analysis were defined for the purpose of the study. The sum of five selected biochemical and physiological traits influencing soybean and common bean productivity was captured in the database (Table 4). Chlorophyll content reflects the plants’ ability to perform photosynthesis efficiently, which is essential for growth and yield. Photosynthesis is quantified by the rate of carbon dioxide (CO_2_) assimilation, representing the plants’ ability to convert light energy into chemical energy. Proline levels serve as a marker for osmotic adjustment and stress tolerance, while catalase and superoxide dismutase are antioxidant enzymes measured to assess the plants’ ability to mitigate oxidative stress. The selected biochemical and physiological traits are essential for plant growth, stress tolerance, and productivity. Chlorophyll content and photosynthesis control how plants capture and use energy, directly affecting biomass production and yield [79,80]. Proline helps plants survive stress by protecting cells, stabilizing proteins, and maintaining water balance [81,82]. Additionally, antioxidant enzymes like catalase and superoxide dismutase play a crucial role in neutralizing reactive oxygen species, preventing cell damage, and ensuring normal growth and development even under stress conditions [83]. These five traits comprehensively capture priming’s impact on photosynthetic performance, osmotic regulation, and antioxidant defense, which are the primary mechanisms driving crop productivity [84].

### 2.4. Data Analysis

Summary statistics, including minimum, maximum, mean, median, and quartile values, were calculated as to assess variability across variables. Outliers were removed before analysis, to improve accuracy, enhance model performance, and prevent misleading conclusions by ensuring that the data accurately represents the population of interest. Box plots were then created in IBM SPSS software version 30.0 to visualize the distribution of data for each trait, including the minimum, maximum, median, and quartile values. Effect estimates, forest plots, and effect sizes were generated using R for meta-analytic synthesis of priming effects. Spearman Rank correlation was further used to determine the magnitude of associations between variables. A biplot principal component analysis (PCA) was conducted using R statistical software (R4.4.3) to show the multiple relationships of the variation for climatic conditions, priming agents, crop type, soil properties, and physiological and biochemical traits.

## 3. Results

### 3.1. Study-Level Effect Estimates

Plots of individual study effect estimates revealed substantial variability in seed priming responses across both physiological and biochemical traits (Figures S1–S5). Chlorophyll content exhibited predominantly positive study-level effects, with a pooled random-effects mean difference (MD) of 21.89 (95% CI: 8.21–35.57; Q = 98,587.84, *p* < 0.001; I^2^ = 100%) (Figure S1). Net photosynthesis showed uniformly positive estimates, yielding a pooled MD of 25.15 (95% CI: 12.63–37.67; Q = 3265.08, *p* < 0.001; I^2^ = 99.9%) (Figure S2). Proline responses displayed extreme dispersion, ranging from strongly negative to highly positive values, with a pooled MD of 19.69 (95% CI: −15.05–54.42; Q = 165,826.17, *p* < 0.001; I^2^ = 100%), indicating a non-significant overall effect (Figure S3). Superoxide dismutase activity demonstrated predominantly positive study-level estimates, with a pooled MD of 26.44 (95% CI: 14.98–37.90; Q = 63,168.85, *p* < 0.001; I^2^ = 100%) (Figure S4). Similarly, catalase activity showed a pooled MD of 24.54 (95% CI: 13.03–36.04; Q = 37,495.00, *p* < 0.001; I^2^ = 100%) (Figure S5).

### 3.2. Impact of Crop Type on Physiological and Biochemical Responses

**Figure 2:**
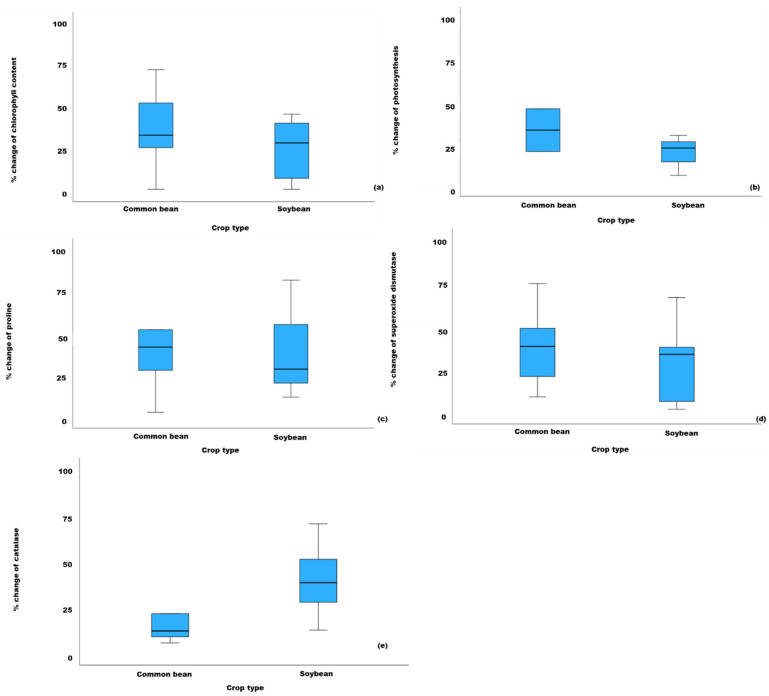
Distribution of physiological and biochemical responses of common bean and soybean to seed priming: (**a**) chlorophyll content (CC), (**b**) net photosynthetic rate (Pn), (**c**) proline accumulation (Pro), (**d**) superoxide dismutase (SOD) activity, and (**e**) catalase (CAT) activity. Each box displays the minimum, maximum, median, and interquartile distribution (25th to 75th percentile) of the percentage changes observed in response to priming.

Common bean exhibited higher mean percentage changes in chlorophyll content (CC) (36.57%), net photosynthetic rate (Pn) (33.17%), proline accumulation (Pro) (45.17%), and superoxide dismutase (SOD) activity (37.10%), as compared to soybean, which recorded respective mean changes of 25.60%, 19.80%, 40.91%, and 30.46%, respectively (Figure 2). The mean percentage changes in chlorophyll content and net photosynthesis were 10.97 and 13.37 percentage points higher, respectively, in common bean than in soybean. Similarly, proline and superoxide dismutase levels were 4.26 and 6.64 percentage points higher in common bean. Soybean exhibited higher catalase activity, with a mean percentage increase of 40.05%, which was 20.56 percentage points higher than that of common bean, with a value of 19.49%.

### 3.3. Impact of Priming Agent on Physiological and Biochemical Responses to Seed Priming

**Figure 3:**
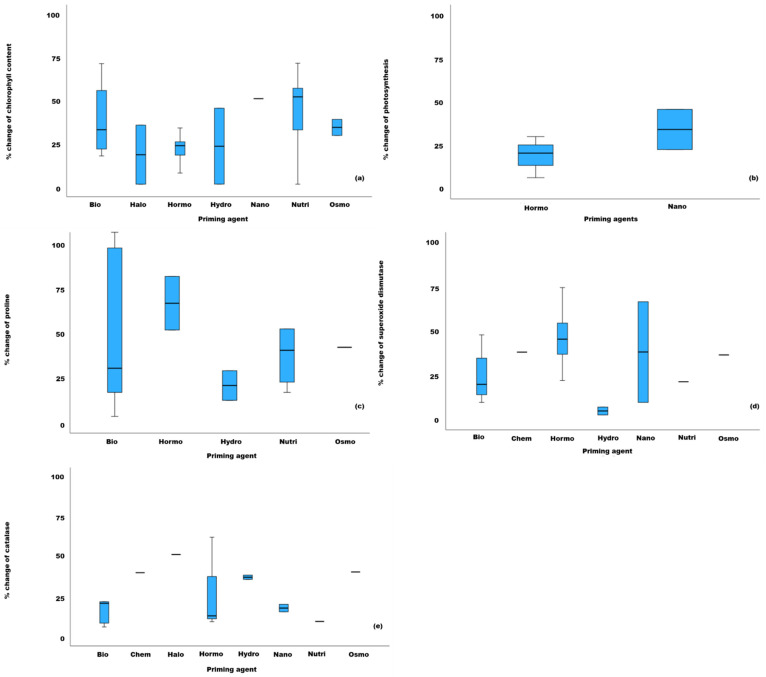
Effects of different seed priming agents on physiological and biochemical responses in common bean and soybean. (**a**) Chlorophyll content (CC), (**b**) net photosynthetic rate (Pn), (**c**) proline accumulation (Pro), (**d**) superoxide dismutase (SOD) activity, and (**e**) catalase (CAT) activity. Each box plot displays the minimum, maximum, median, and interquartile distribution (25th to 75th percentile) of the percentage changes observed in response to priming.

Chlorophyll content (%CC) showed the highest mean percentage increase under nutri-priming at 42.44% (range: 1.67–71.70%), followed by bio-priming at 38.98% (range: 18.02–71.43%). Halo-priming and hydro-priming recorded lower mean percentage changes of 18.77% and 23.67%, respectively, while nano-priming showed no variation in %CC (Figure 3). For net photosynthesis (%Pn), nano-priming exhibited the highest mean percentage change of 34.18% (range: 22.68–45.69%), whereas hormonal priming had the lowest change at 19.12% (range: 6.61–30.09%). Proline accumulation (%Pro) showed the largest mean percentage increase with hormonal priming at 66.27% (range: 51.43–81.12%), closely followed by bio-priming at 65.80% (range: 3.68–163.33%). Nutri-priming and hydro-priming exhibited lower mean percentage changes of 33.31% and 20.82%, respectively, while osmo-priming showed no variation in %Pro. The mean percentage changes in superoxide dismutase activity (%SOD) were highest under hormonal priming (47.45%; range: 22.19–74.40%), followed by nano-priming (38.15%; range: 9.89–66.42%) and bio-priming. Nutri-priming and hydro-priming recorded the lowest mean percentage changes at 22.53% and 20.82%, respectively, with no variation in %SOD observed under osmo-priming. Catalase activity (%CAT) displayed a unique trend, with the highest mean percentage change recorded for hydro-priming (37.24%; range: 35.90–38.58%), followed by hormonal priming (28.32%; range: 9.61–62.15%) and bio-priming. Nano-priming exhibited the lowest mean percentage changes at 18.04% (range: 15.74–20.36%). Notably, %CAT showed no variation under chemical, nutri-, halo-, or osmo-priming.

### 3.4. Impact of Climatic Conditions on Physiological and Biochemical Responses to Seed Priming

**Figure 4:**
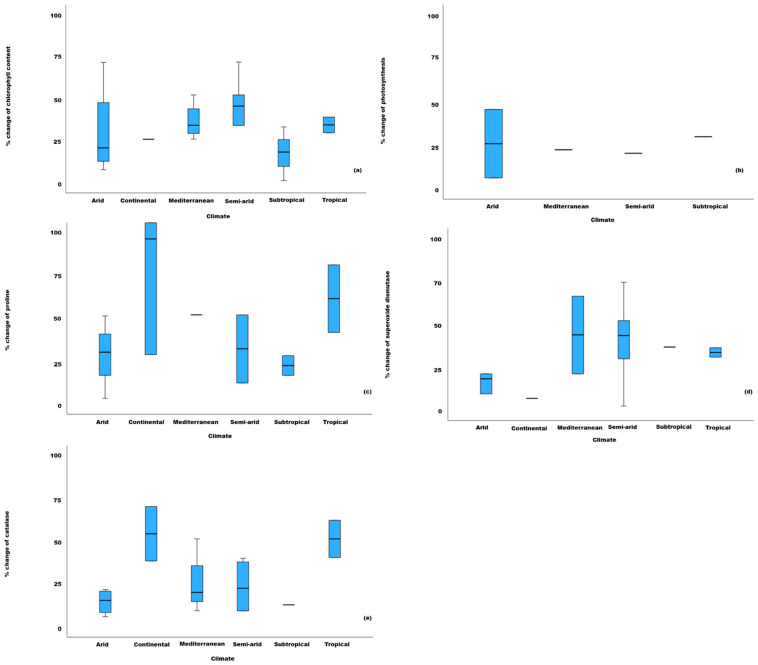
Effects of different climatic changes on physiological and biochemical responses to seed priming in common bean and soybean. The box plots represent changes in (**a**) chlorophyll content (CC), (**b**) photosynthetic rate (Pn), (**c**) proline accumulation (Pro), (**d**) superoxide dismutase (SOD) activity, and (**e**) catalase (CAT) activity. Each box plot displays the minimum, maximum, median, and interquartile distribution (25th to 75th percentile) of the percentage changes observed in response to priming.

The highest mean percentage increase in chlorophyll content (%CC) was observed in the semi-arid climate (40.1%; range: 1.67–71.70%), followed by the Mediterranean (36.8%; range: 26.22–52.21%) and tropical (34.6%; range: 29.92–39.21%) climates. In contrast, the lowest %CC means were recorded in the arid (30.4%; range: 8.16–71.4%) and subtropical (21.72%; range: 1.68–33.33%) climates, while %CC remained constant in the continental climate (Figure 4). The highest mean percentage increase in net photosynthesis (%Pn) was found in the arid climate, with a mean of (26.15%; range: 6.61–45.69%), while %Pn remained constant in the Mediterranean, semi-arid, and subtropical climates. Proline accumulation (%Pro) exhibited a different trend, with the continental climate displaying the highest mean percentage increase (96.18%; range: 29.02–163.33%), followed by the tropical (61.48%; range: 41.84–81.12%) and semi-arid (32.34%; range: 12.61–52.07%) climates. The subtropical (20.79%; range: 17.01–28.35%) and arid (8.49%; range: 3.68–51.43%) climates recorded the lowest %Pro means. Superoxide dismutase activity (%SOD) was highest in the Mediterranean climate, with a mean of 43.97% (range: 21.53–66.42%), followed closely by the semi-arid (42.63%; range: 2.86–74.40%) and tropical (33.82%; range: 31.13–36.51%) climates. The lowest %SOD means were reported for the arid climate (21.54%; range: 9.89–47.76%), while %SOD remained unchanged in the continental and subtropical climates. Catalase activity (%CAT) showed a distinct pattern, with the highest mean percentage increase recorded in the continental climate (54.34%; range: 38.59–70.10%), followed by the tropical (51.36%; range: 40.58–62.15%) and Mediterranean (27.17%; range: 9.76–51.39%) climates. Lower %CAT means were observed in the semi-arid (23.83%; range: 9.62–40.08%) and arid (14.76%; range: 6.32–22.00%) climates, while %CAT remained unchanged in the subtropical climate (Figure 4 and Figure S14).

### 3.5. Correlation Analysis: Associations Between Physiological and Biochemical Traits, and Environmental and Soil Factors

Clay content showed a positive correlation with chlorophyll content (%CC) (r = 0.49, *p* < 0.05) and catalase activity (%CAT) (r = 0.58, *p* < 0.05). Potassium (K) had a positive correlation with proline (%Pro) (r = 0.64, *p* < 0.05) and a highly significant positive correlation with superoxide dismutase (%SOD) (r = 0.57, *p* < 0.01). pH was negatively correlated with %Pro (r = −0.64, *p* < 0.05). Bulk density (BD) showed a positive correlation with photosynthesis percentage (%Pn) (r = 0.90, *p* < 0.05). Sand content was negatively correlated with %CC (r = −0.48, *p* < 0.05). Priming agent showed a positive correlation with catalase activity (%CAT), whilst photosynthesis (%Pn) and superoxide dismutase (%SOD) showed a positive correlation with crop type.

### 3.6. Principal Component Analysis

**Figure 5:**
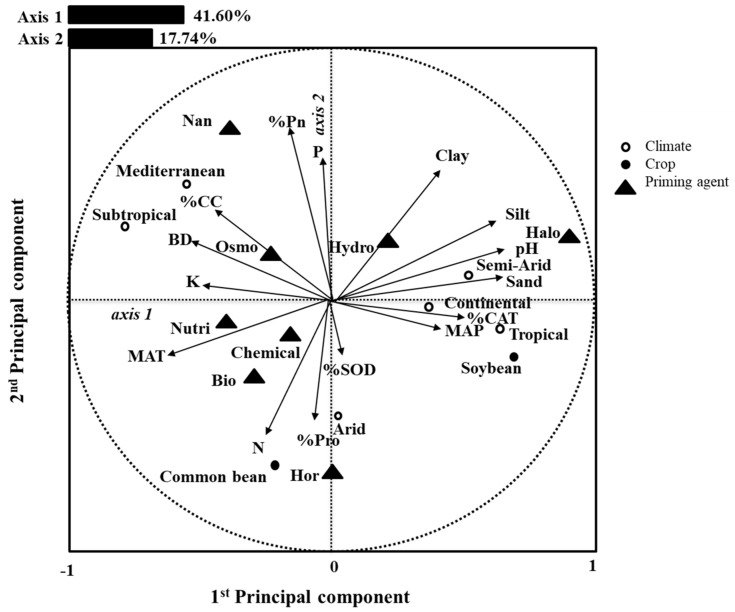
Principal component analysis (PCA) showing the relationship among environmental factors, soil properties, climatic conditions, priming agents, crops, BD, and soil bulk density (g cm^−3^); MAP, mean annual precipitation; MAT, mean annual temperature.

The interrelationships among the assessed parameters (climate conditions, crops, priming agents, soil properties, and physiological and biochemical traits) were explored using principal component analysis (PCA) (Figure 5). The PCA showed that the first principal component (PC1) explains a significant portion of the total variation (41.60%) and exhibited a higher positive association with bulk density (BD) and soil potassium (K). The second principal component (PC2) accounted for a substantial percentage (17.74%) of the data variation and had a higher positive correlation with phosphorus (P) and soil pH, while showing a negative correlation with variables such as superoxide dismutase (%SOD) and proline (%Pro). Common bean was positioned closer to nitrogen (N) and hormonal priming, suggesting a strong interaction between these variables. Conversely, soybean aligned with continental and tropical climates, along with catalase activity (%CAT) and mean annual precipitation (MAP), highlighting a different response pattern. Among priming agents, hydro-priming showed a stronger association with soil phosphorus, while halo-priming was linked to soil pH and silt content. Osmo-priming correlated positively with bulk density and chlorophyll content (%CC), whereas hormonal priming aligned with antioxidant responses such as %SOD and %Pro.

## 4. Discussions

### 4.1. Study-Level Effect Estimates

The extremely high heterogeneity (I^2^ ≈ 99–100%) observed across all traits indicates substantial variability in seed priming responses among studies, reflecting the complex and multifactorial nature of plant responses to priming. Seed priming outcomes are shaped by interacting factors, including crop species and cultivar differences, the type and concentration of the priming agent, treatment duration, and the nature, intensity, and timing of stress exposure [85]. These variables can differentially affect photosynthetic performance, osmolyte accumulation, and antioxidant enzyme regulation, leading to a wide dispersion of effect sizes. Biochemical traits, such as proline content and antioxidant enzyme activities, are highly dynamic and particularly sensitive to developmental stage and sampling time, which could further amplify variability across studies [86]. Overall, these patterns highlight that seed priming effects are conditional and should be interpreted within specific ecological and methodological contexts, rather than assumed to be universally consistent.

### 4.2. Physiological and Biochemical Responses to Seed Priming Based on Crop Type

The present study showed that common bean and soybean crops exhibit variations in their physiological and biochemical responses to seed priming (Figure 2). A higher response of the studied variables was noted for common bean, except for catalase activity, whose value was higher for soybean. These differences could be ascribed to the molecular and genetic variations in the two crops [87,88]. Common bean has been found to possess a broader genetic base due to its two distinct domestication gene pools, Mesoamerican and Andean, which provide a wider range of adaptive traits that enhance its ability to respond dynamically to any environmental stimuli, including priming treatments [89,90]. Additionally, the higher total polyphenol contents reported in common bean, compared to other legumes such as chickpea, lentils and soybean may neutralize reactive oxygen species, protecting vital cellular structures, and thus contributing to superior physiological and biochemical responses [91,92]. In contrast, soybean has undergone extensive artificial selection for high oil content and yield, which may lead to a genetic bottleneck that can constrain its physiological flexibility and biochemical resilience [93,94]. Furthermore, soybean has been reported to be more sensitive to over-hydration, which can negatively impact responses if primed improperly [95,96]. These results are consistent with the findings of [97,98], whose studies reported varying results on different crops (including sorghum and barley) that were subjected to the same priming treatment of calcium chloride on the antioxidant activities, with one reporting reduction and enhancement in performance, respectively.

### 4.3. Impact of Priming Agents on Physiological and Biochemical Responses to Seed Priming

Priming agents have a determining factor in the response of the physiological and biochemical responses of plants to seed priming [37,99]. Their efficacy varies and hence there may be specific agents only suitable for specific species, cultivars and variables [100]. According to the results of this study, certain priming agents may have a profound impact on the enhancement of certain parameters but not be as effective for others (Figure 3 and Figures S6–S10). This is due to the fact that different priming agents affect the physiological and biochemical traits in plants differently due to the unique ways they interact with the plant’s cellular processes and stress response mechanisms [39,101]. For instance, some agents may be enhanced more in the areas of water uptake and metabolism [102,103], while others might regulate ion balance, hormonal pathways or enzyme activities [104]. These differences in mechanisms can lead to variations in plant’s responses, leading to the traits more favored by the triggered mechanism showing more pronounced benefits [105,106]. Similar results were reported by Zhu et al. [107], who observed that priming seeds of rapeseed (*Brassica napus* L.) with salicylic acid, gibberellic acid and abscisic acid led to a significant increase in net photosynthesis, stomatal conductance and transpiration rate but led to a decrease in the antioxidant enzyme activities of catalase (CAT) and ascorbic peroxidase (APX) compared to the control. Similarly, Chen et al. [97] also reported that priming sorghum seeds with calcium chloride improved germination, osmotic regulation and ion balance but decreased antioxidant enzyme activities along with related gene expressions compared to unprimed seeds.

### 4.4. Impact of Climatic Conditions on Physiological and Biochemical Responses to Seed Priming

The physiological and biochemical responses to seed priming varied across climatic conditions (Figures S11–S15). A higher response for physiological parameters was noted for harsher conditions (arid and semi-arid), whereas for biochemical parameters, a higher response was shown for moderate or conducive conditions (continental and Mediterranean climates) (Figure 4). The observed difference in priming responses likely results from the distinct adaptive demands placed on plants by varying climatic stressors. Harsh environments such as arid and semi-arid climates are characterized by severe water deficits, extreme temperatures, and high radiation levels [108,109]. Under such conditions, plants may prioritize physiological traits like maintaining chlorophyll content and optimizing photosynthesis, as these processes are essential for energy acquisition and survival [110]. Chlorophyll preservation ensures the continuation of light absorption, while photosynthesis underpins energy production, crucial for sustaining growth and combating environmental stress [111,112]. These adaptations are immediate and directly linked to survival under acute stress [113,114]. In contrast, conducive climates like continental and Mediterranean regions impose less extreme but more chronic or predictable stressors, such as oxidative challenges caused by seasonal temperature fluctuations or moderate droughts [115]. In such environments, plants rely heavily on biochemical responses, such as the accumulation of proline and the activation of antioxidant enzymes like superoxide dismutase and catalase [116]. Proline functions as an osmo-protectant, stabilizing cellular structures and maintaining osmotic balances, while SOD and CAT play a vital role in scavenging reactive oxygen species (ROS), thereby preventing oxidative damage to cellular components [117,118]. These biochemical responses reflect a more systemic and long-term approach to stress mitigation, enabling the plants to maintain cellular homeostasis and enhance resilience over time [119,120].

### 4.5. The Association Between Crop Type, Priming Agents, Climatic Conditions and Soil Properties Towards Physiological and Biochemical Responses of the Crops

The degree of correlation between crop type, priming agents, climatic conditions, and soil properties with physiological and biochemical traits in common bean and soybean provides valuable insights for optimizing crop management practices (Table 5). A strong correlation was observed between crop type and traits like photosynthesis and superoxide dismutase activity, underscoring the role of genetic diversity among species in influencing the plants’ trait responses to seed priming [121]. Additionally, a positive correlation between priming agents and catalase activity suggests that priming agents may enhance the plants’ antioxidant defense, likely because they have been found to be effective in preparing plants for stress conditions through improving the enzyme activations [122]. The positive association observed between clay content and traits such as chlorophyll content and catalase activity suggests that soils with higher clay content may create a more favorable environment for stress-related enzymes and nutrient retention [123]. In contrast, the negative correlation of sandy soils with chlorophyll content indicates the limitations of such soil types in supporting optimal plant growth [124,125]. Potassium also showed a positive relationship with proline and antioxidant activities (SOD and CAT), which may underscore its critical role in enhancing stress resilience and metabolic stability [126,127].

The PCA biplot (Figure 5) reveals the significant role of factors such as crop type, climatic conditions, soil properties, and priming agents in shaping the plants’ physiological and biochemical responses to seed priming. Soybean’s close association with variables like clay, silt, pH, tropical conditions, and catalase (CAT) activity suggests that the crop might thrive in environments with finer soil textures and stable pH levels [128]. On the other hand, common bean exhibits clustering with variables such as nitrogen, arid conditions, superoxide dismutase (SOD) activity, and proline, which signifies its enhanced response to seed priming in drier environments with elevated nitrogen levels [129].

### 4.6. Cis- and Trans-Priming (Cross-Tolerance) in Seed Priming Responses

Cis-priming represents stimulus-specific defense activation where the priming agent directly triggers responses precisely matched to the encountered stress, while trans-priming confers cross-tolerance by elevating the plant’s baseline immunity to protect against diverse, unrelated stresses beyond the initial priming stimulus [130]. Adaptive demands of certain conditions may preferentially activate one mechanism over the other, as shown in Figure 4, where harsher arid/semi-arid environments favor cis-priming to rapidly enhance physiological responses critical for immediate survival, while moderate continental/Mediterranean climates promote trans-priming to strengthen biochemical resilience against chronic stressors.

These mechanisms operate through coordinated transcriptome reprogramming, where cis-priming rapidly upregulates stress-specific genes (ABA-responsive elements and DREB factors) to drive physiological execution, while trans-priming activates broader defense networks (SA/JA pathways and PTI-related genes) for systemic biochemical enhancement [131]. This molecular coordination directly explains the observed physiological and biochemical responses in this study, demonstrating seed priming’s inherently multi-level nature that simultaneously integrates physiological trait modification, molecular signaling coordination, and immunological memory formation to optimize stress adaptation across diverse crops, environments, and treatments.

## 5. Conclusions

This study analyzed 31 research articles to evaluate how climatic conditions, crop type, and priming agents affect the physiological and biochemical responses of common bean and soybean to seed priming, revealing stronger overall responses in common bean, except for higher catalase activity in soybean, with variations across priming agents and climates. Moreover, seed priming operates through cis- and trans-priming mechanisms, functioning as a multi-level preconditioning strategy where cis-priming drives rapid stimulus-specific physiological adjustments, while trans-priming establishes broader biochemical and immunological resilience. Despite these insights, several limitations should be acknowledged. The current review’s focus on overall priming capacity rather than explicitly separating responses to different stress types (e.g., drought, salinity, and heat) represents a key limitation, as it precludes stress-specific priming recommendations and obscures mechanism–stress interactions critical for targeted applications. Additionally, inconsistent reporting of soil moisture dynamics, temperature extremes, and microbial activity across studies further constrains ecological interpretation; therefore, future research should standardize these variables. Region-specific reviews targeting zones are also recommended to develop optimized priming protocols adapted to local environmental conditions.

## Figures and Tables

**Table 2:** Description of environmental variables used in the study.

Environmental Factors	Symbols	Units	Definitions
Mean annual temperature	MAT	°C yr^−1^	The average temperature over a year for the study area.
Mean annual precipitation	MAP	mm yr^−1^	The average amount of rainfall and other precipitation that falls over the study area in one year.
Soil pH	pH		The topsoil pH (0–30 cm) reported in studies for the study area.
Soil bulk density	BD	g cm^−3^	The bulk density of the topsoil layer (0–30 cm).
Clay content	Clay	%	The average clay content (fine-textured soil particles) of the topsoil layer (0–30 cm).
Sand content	Sand	%	The average sand content (coarse-textured soil particles) of the topsoil layer (0–30 cm).
Silt content	Silt	%	The average silt content (medium-textured soil particles) of the topsoil layer (0–30 cm).
Nitrogen	N	mg kg^−1^	The nitrogen content of the topsoil layer (0–30 cm), as reported in papers.
Phosphorus	P	mg kg^−1^	The phosphorus content of the topsoil layer (0–30 cm), as reported in the papers.
Potassium	K	mg kg^−1^	The potassium content of the topsoil layer (0–30 cm), as reported in the layer.

**Table 3:** List of classes describing the environmental factors, remarks, ranges and classifications.

Environmental Factors	Remarks	Ranges	Classifications
Soil pH	The topsoil pH (0–30 cm) reported in studies for the study area.	<5.5	Highly acidic
		5.5–6.5	Acidic
		6.5–7.5	Neutral
		7.5–8.5	Basic (alkaline)
		>8.5	Highly Basic (Strongly alkaline)
Sand %	The average sand content (coarse-textured soil particles) of the topsoil layer (0–30 cm).	Less than 20%	Low
		20–50%	Medium
		Above 50%	High
Clay %	The average clay content (fine-textured soil particles) of the topsoil layer (0–30 cm).	Less than 20%	Low
		20% to 35%	Medium
		More than 35%	High
Silt %	The average silt content (medium-textured soil particles) of the topsoil layer (0–30 cm).	Less than 20%	Low
		20% to 40%	Medium
		More than 40%	High
Soil bulk density	The bulk density of the topsoil layer (0–30 cm).	Less than 1.3 g/cm^3^	Low
		1.3 to 1.6 g/cm^3^	Medium
		More than 1.6 g/cm^3^	High
Climatic regions	Hot and Wet	MAP: >2000 mm yr^−1^ MAT: 18–28 °C yr^−1^	Tropical
	Warm and Wet	MAP: 1000–2000 mm yr^−1^ MAT: 15–18 °C yr^−1^	Subtropical
	Cool and Moist	MAP: 500–1000 mm yr^−1^ MAT: 5–15 °C yr^−1^	Temperature
	Hot and Dry	MAP: <250 mm yr^−1^ MAT: >18 °C yr^−1^	Arid
	Warm and dry	MAP: 250–500 mm yr^−1^ MAT: 18–25 °C yr^−1^	Semi-arid
	Warm and Dry (Wet Winters)	MAP: 400–800 mm yr^−1^ MAT: 10–20 °C yr^−1^	Mediterranean
	Hot Summers and Cold Winters	MAP: 300–800 mm yr^−1^ MAT: −3–18 °C yr^−1^	Continental

**Table 4:** Definition of physiological and biochemical traits used in this study.

Variable	Symbol	Units	Definition
Chlorophyll content	CC	mg/m^2^	Refers to the amount of chlorophyll in plant tissues, which is essential for photosynthesis and indicates the plant’s ability to capture light energy.
Photosynthesis	Pn	µmol/m^2^/s	Process by which plants use sunlight to convert carbon dioxide and water into glucose and oxygen.
Proline	Pro	µmol/g FW	Unique amino acid with a distinctive cyclic structure, playing a crucial role in protein synthesis and stability.
Superoxide dismutase	SOD	U/mg protein	Enzyme that protects cells by converting harmful superoxide radicals into oxygen and hydrogen peroxide, thereby mitigating oxidative stress.
Catalase	CAT	U/mg protein	Enzyme that decomposes hydrogen peroxide into water and oxygen, protecting cells from oxidative damage.

**Table 5:** Correlations showing the relationship between physiological and biochemical traits, and environmental and soil factors.

Variables	%CC	%Pn	%Pro	%SOD	%CAT
PH	0.24	−0.80	−0.64 *	−0.17	0.12
BD	−0.17	0.90 *	0.14	−0.34	−0.28
N	−0.21	−0.20	−0.06	−0.37	−0.05
P	0.23	−0.10	0.34	−0.26	−0.10
K	0.37	0.70	0.64 *	0.57 **	0.12
Clay	0.49 *	0.60	0.10	−0.23	0.58 *
Silt	0.45 *	−0.20	−0.27	0.26	0.28
Sand	−0.48 *	−0.70	0.11	0.15	−0.29
Priming agent	−0.23	−0.50	−0.26	0.01	0.54 *
Crop type	−0.12	0.90 *	0.09	0.52 *	−0.36
Climate	−0.11	0.50	−0.37	0.14	−0.19

Note: (*) denotes significance (*p* < 0.05); (**) denotes significance (*p* < 0.01).

## Data Availability

The datasets generated and analyzed during the present study are available from the corresponding author upon request. The data are not publicly available due to confidentiality and privacy considerations.

## Supplementary Materials

The following supporting information can be downloaded at: https://www.mdpi.com/article/10.3390/plants15111636/s1. Reference [132] is cited in the Supplementary Materials.
