# Supplementary material for: Physiological and Biochemical Responses of Two Major Legume Crops to Seed Priming—A Systematic Review
Source: Plants (Basel). 2026 May 26;15(11):1636. doi: 10.3390/plants15111636 (PMC13259165; doi:10.3390/plants15111636)
Supplement: Supplementary file 1 [file plants-15-01636-s001.zip › plants-4168276-supplementary figures.pdf]

## Supplementary Material

### Supplementary Figures

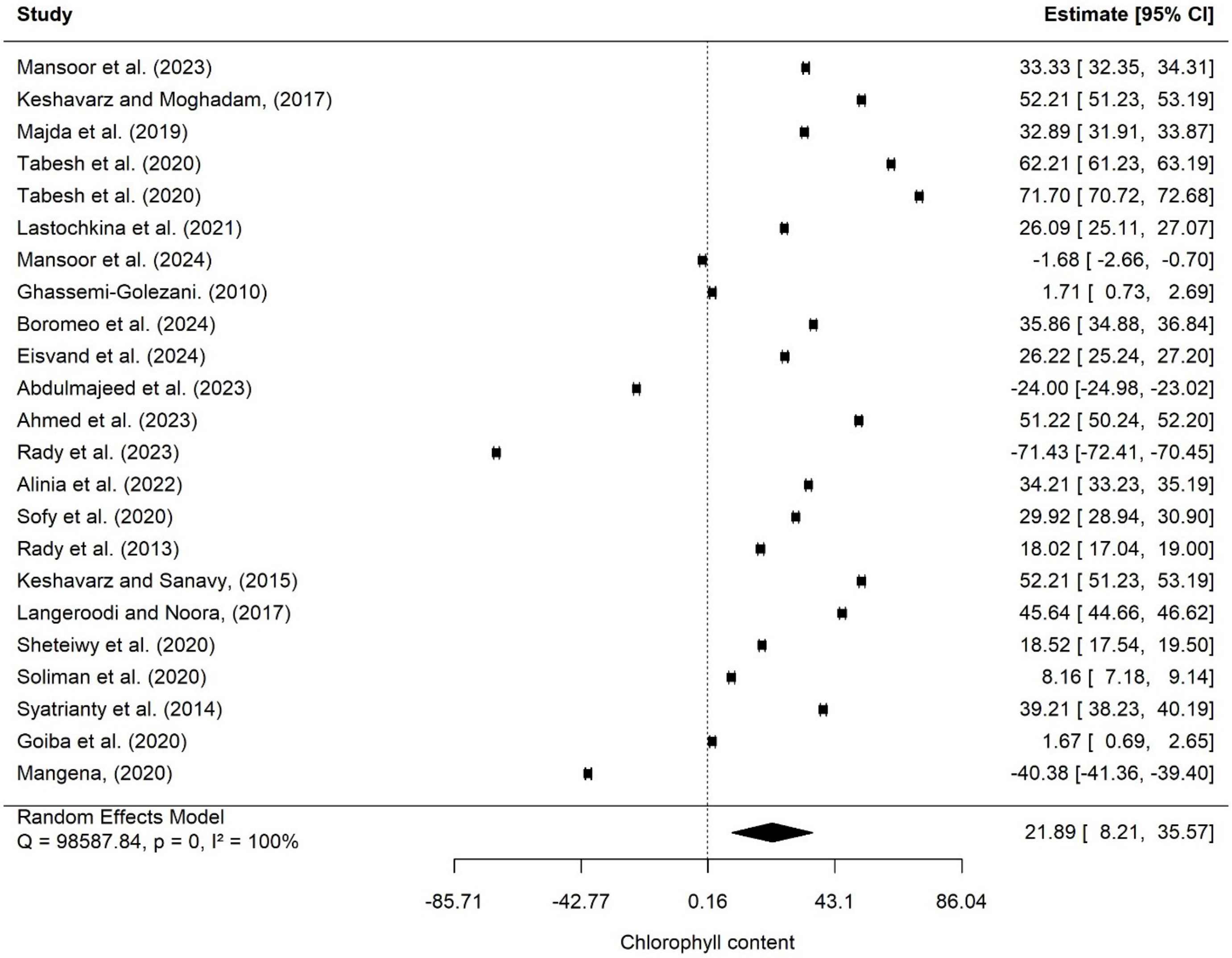


**Supplementary Figure S1:** Plot of individual study effect estimates for chlorophyll content. Filled markers represent study-specific effect sizes, with horizontal lines indicating 95% confidence intervals (CIs); square size reflects study weight. The vertical dashed line denotes the null effect value. The pooled estimate, calculated using a random-effects model, is shown as a diamond, with its width representing the 95% CI. Between-study heterogeneity statistics (Q and $I^2$) are presented below the plot.

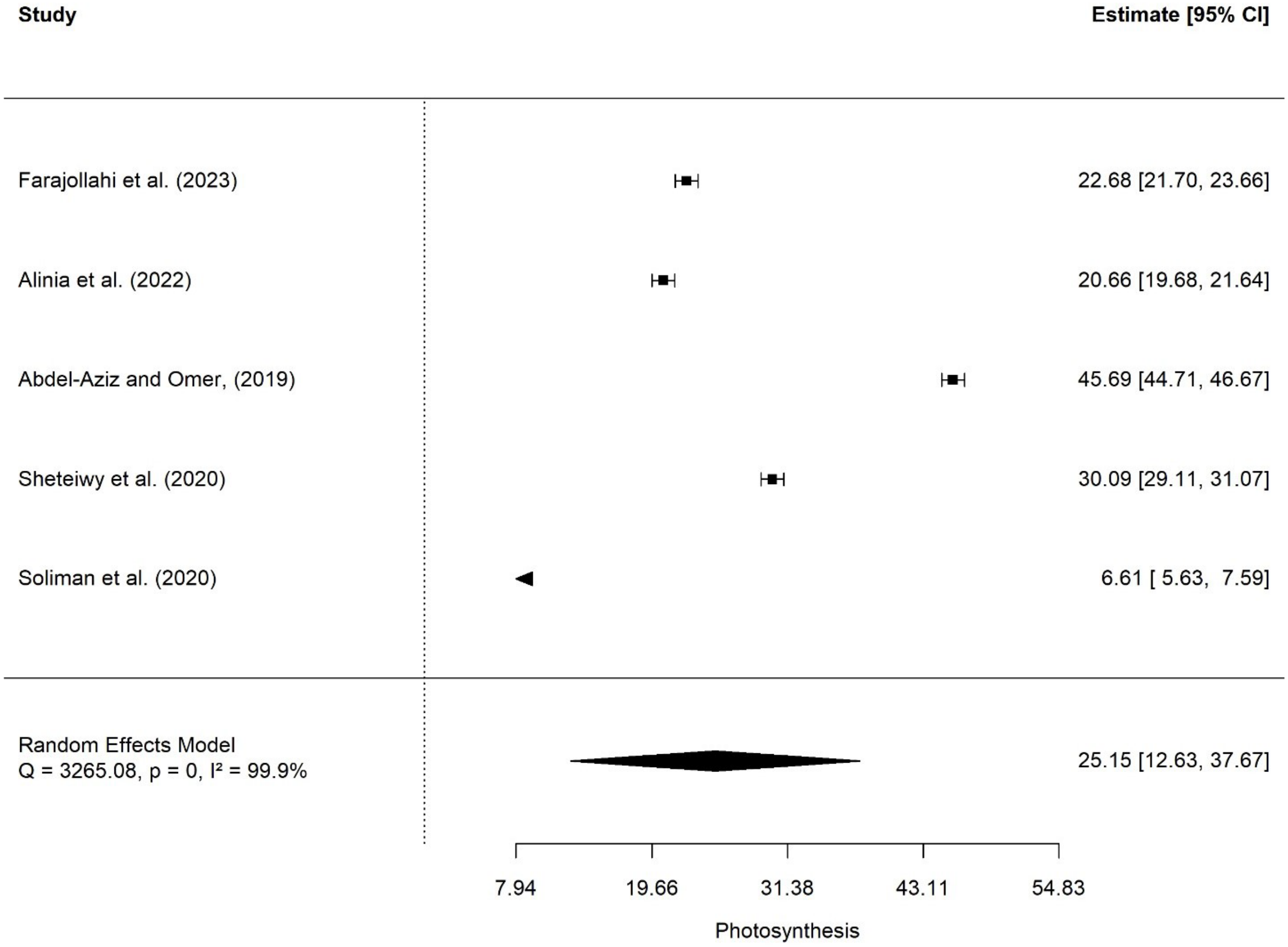


**Supplementary Figure S2:** Plot of individual study effect estimates for photosynthesis. Filled markers represent study-specific effect sizes, with horizontal lines indicating 95% confidence intervals (CIs); square size reflects study weight. The vertical dashed line denotes the null effect value. The pooled estimate, calculated using a random-effects model, is shown as a diamond, with its width representing the 95% CI. Between-study heterogeneity statistics (Q and $I^2$) are presented below the plot.

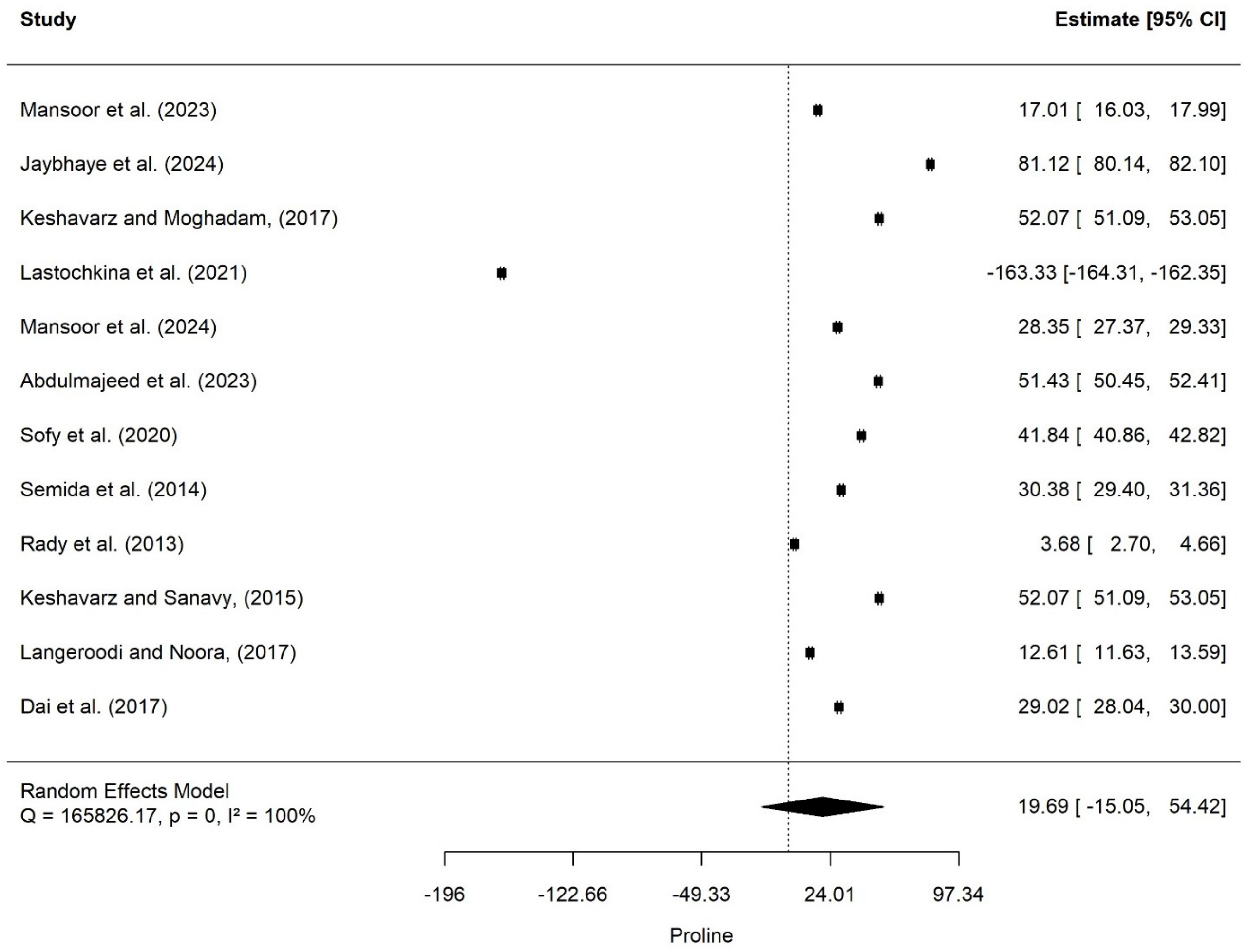


**Supplementary Figure S3:** Plot of individual study effect estimates for proline. Filled markers represent study-specific effect sizes, with horizontal lines indicating 95% confidence intervals (CIs); square size reflects study weight. The vertical dashed line denotes the null effect value. The pooled estimate, calculated using a random-effects model, is shown as a diamond, with its width representing the 95% CI. Between-study heterogeneity statistics (Q and $I^2$) are presented below the plot.

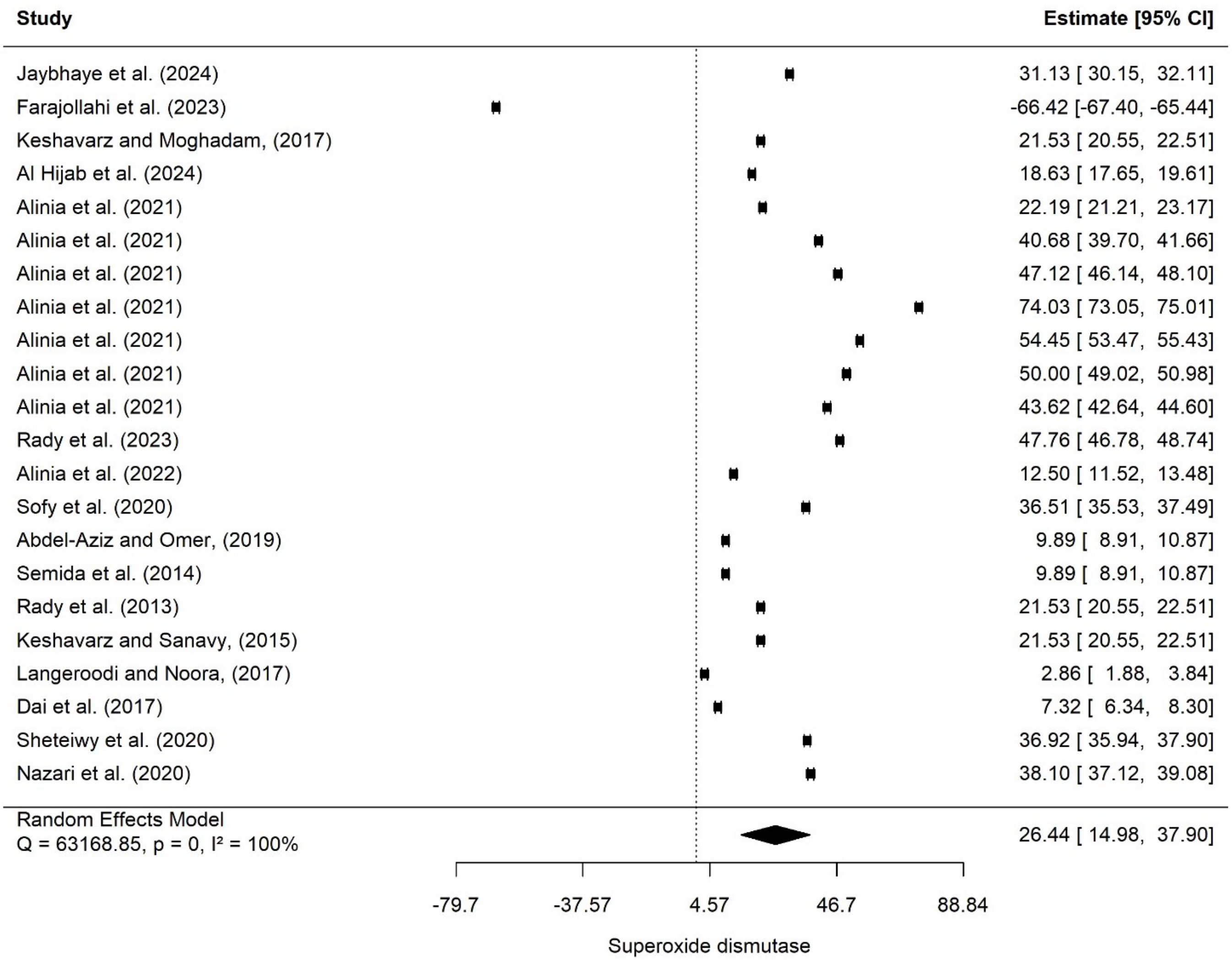


**Supplementary Figure S4:** Plot of individual study effect estimates for superoxide dismutase. Filled markers represent study-specific effect sizes, with horizontal lines indicating 95% confidence intervals (CIs); square size reflects study weight. The vertical dashed line denotes the null effect value. The pooled estimate, calculated using a random-effects model, is shown as a diamond, with its width representing the 95% CI. Between-study heterogeneity statistics (Q and $I^2$) are presented below the plot.

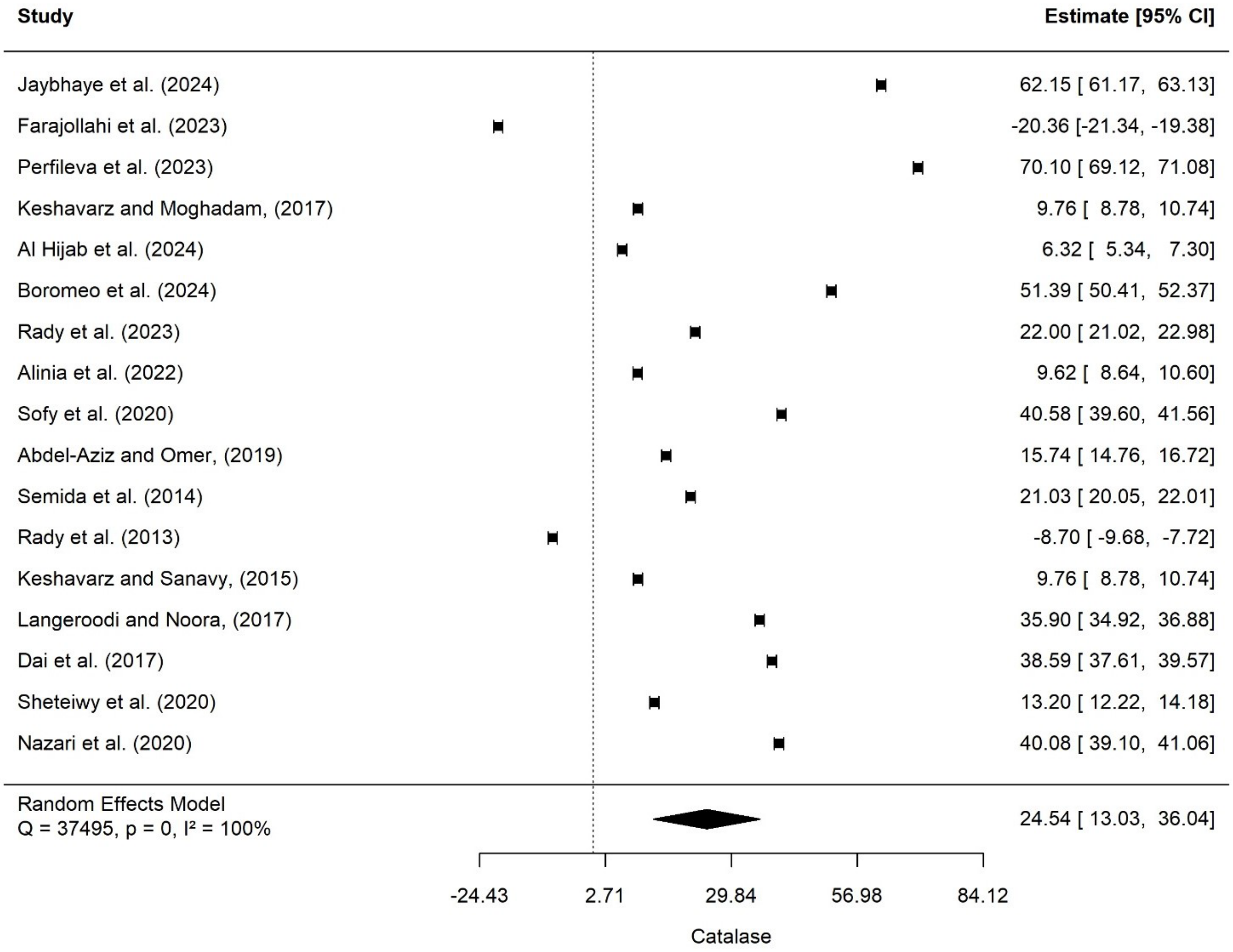


**Supplementary Figure S5:** Plot of individual study effect estimates for catalase. Filled markers represent study-specific effect sizes, with horizontal lines indicating 95% confidence intervals (CIs); square size reflects study weight. The vertical dashed line denotes the null effect value. The pooled estimate, calculated using a random-effects model, is shown as a diamond, with its width representing the 95% CI. Between-study heterogeneity statistics (Q and $I^2$) are presented below the plot.

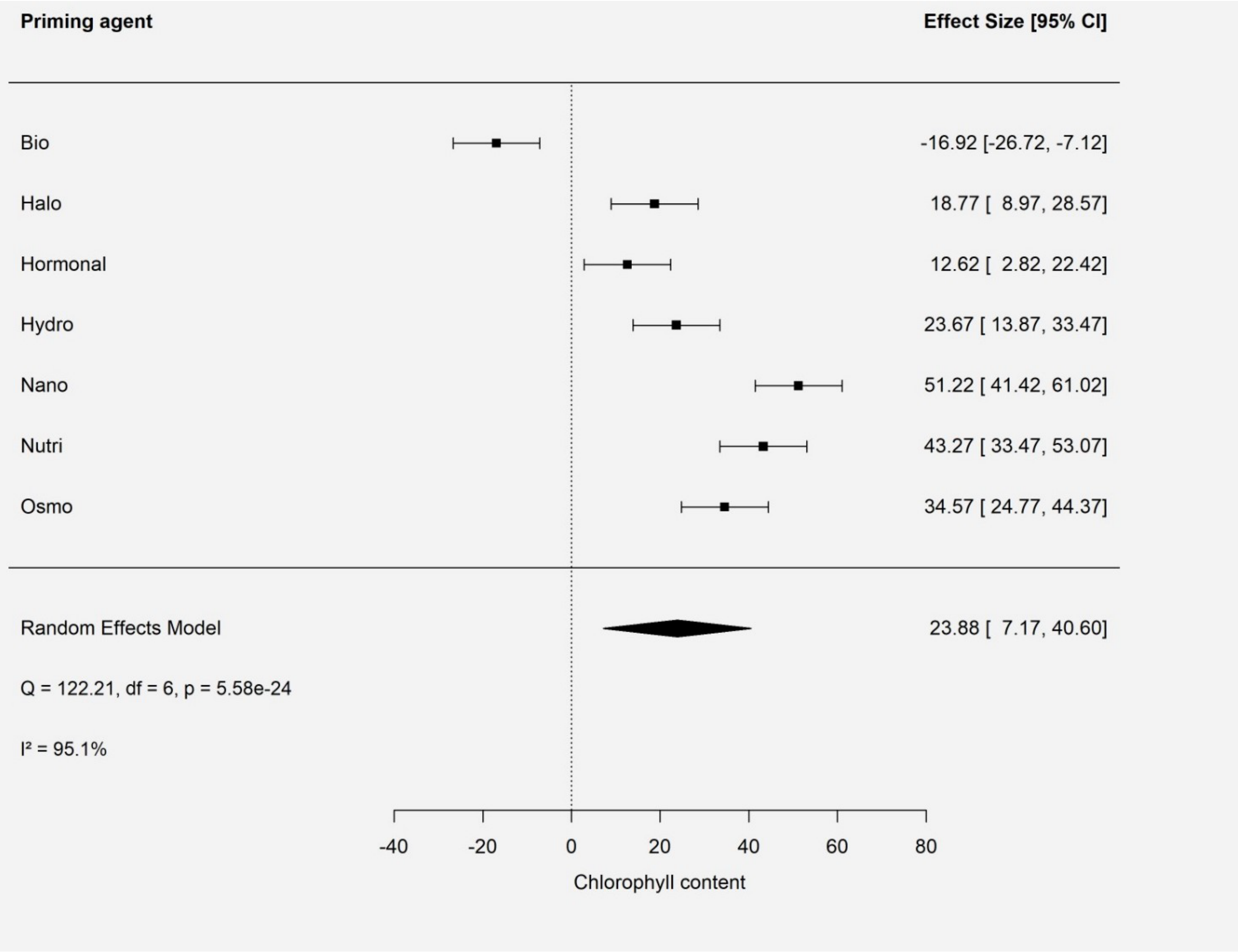


**Supplementary Figure S6:** Effect of different priming agents on chlorophyll content. Plot showing the effect sizes (mean differences) and 95% confidence intervals (CI) for chlorophyll content following treatment with priming agents: Bio, Halo, Hormonal, Hydro, Nano, Nutri, and Osmo. Negative values indicate a reduction in chlorophyll content relative to control, while positive values indicate an increase. Each black square represents the effect size for an individual priming agent, with horizontal lines representing the 95% CI. The diamond at the bottom represents the pooled effect size from a random-effects model, with width corresponding to the 95% CI. Heterogeneity statistics are shown ($Q = 122.21$, $df = 6$, $p < 5.58e-24$; $I^2 = 95.1\%$).

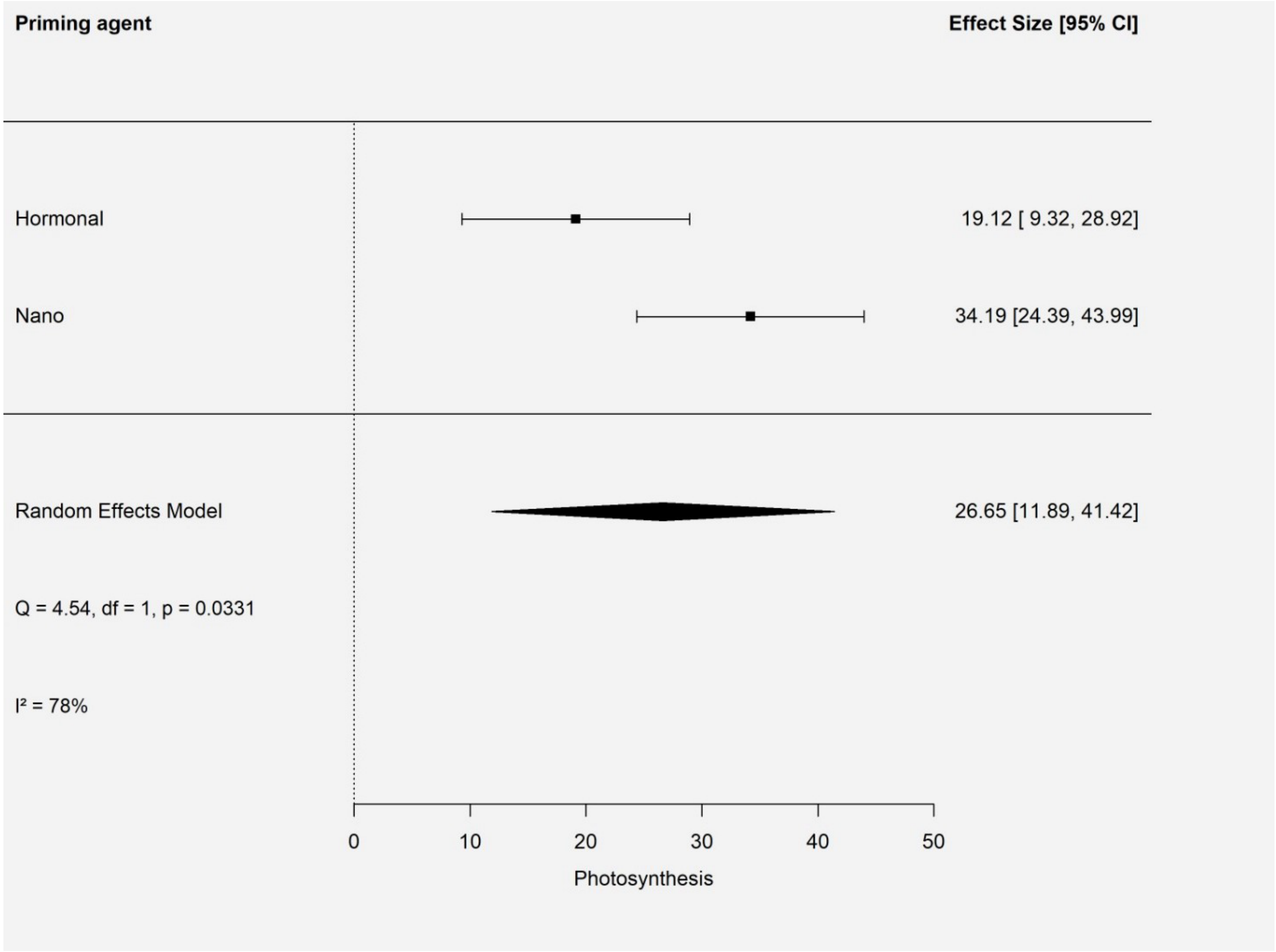


**Supplementary Figure S7:** Effect of different priming agents on photosynthesis. Plot showing the effect sizes (mean differences) and 95% confidence intervals (CI) for photosynthesis following treatment with priming agents: Hormonal, and Nano. Negative values indicate a reduction in photosynthesis relative to control, while positive values indicate an increase. Each black square represents the effect size for an individual priming agent, with horizontal lines representing the 95% CI. The diamond at the bottom represents the pooled effect size from a random-effects model, with width corresponding to the 95% CI. Heterogeneity statistics are shown ($Q = 4.54$, $df = 1$, $p = 0.0331$; $I^2 = 78\%$).

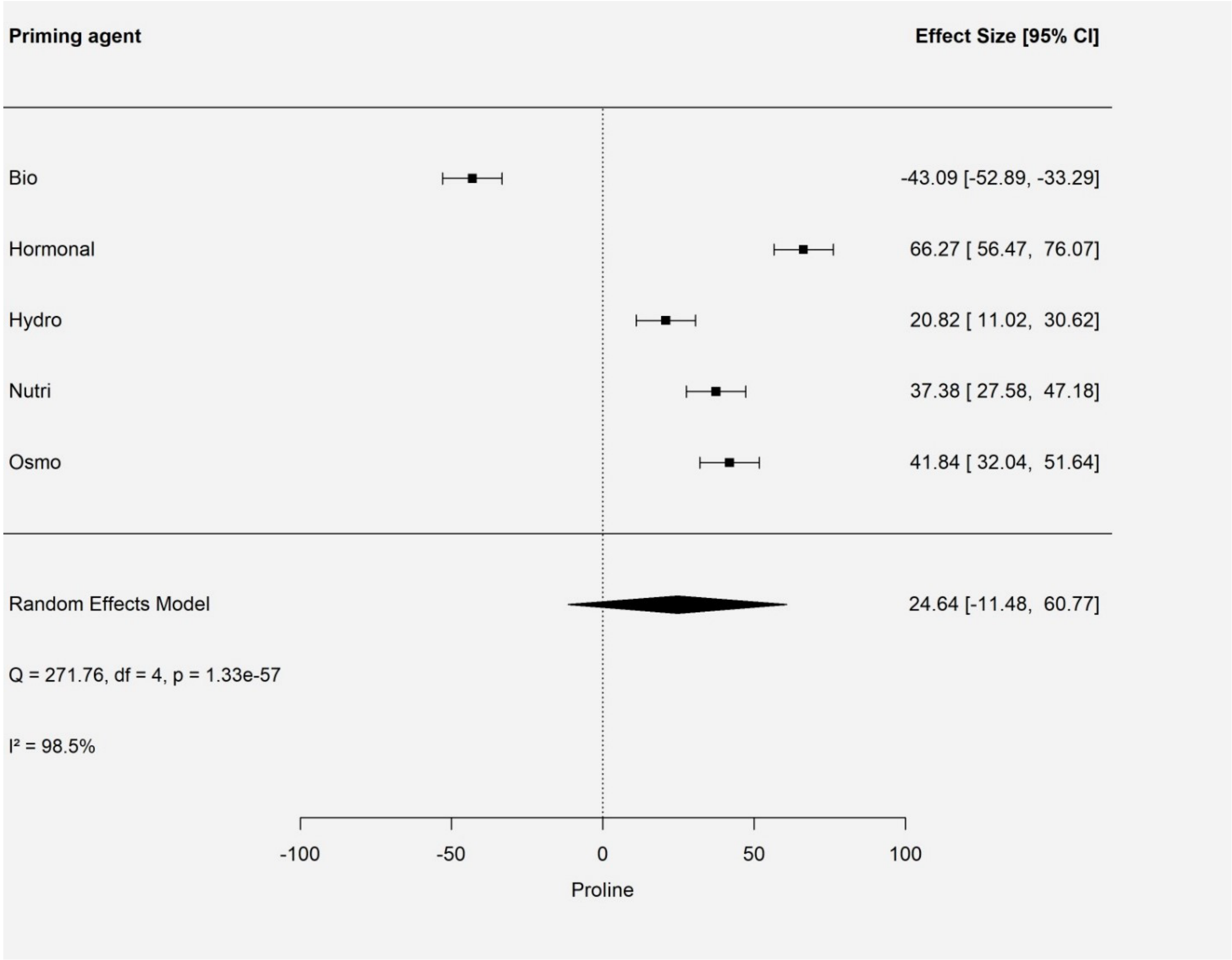


**Supplementary Figure S8:** Effect of different priming agents on proline. Plot showing the effect sizes (mean differences) and 95% confidence intervals (CI) for proline following treatment with priming agents: Bio, Hormonal, Hydro, Nutri, and Osmo. Negative values indicate a reduction in proline relative to control, while positive values indicate an increase. Each black square represents the effect size for an individual priming agent, with horizontal lines representing the 95% CI. The diamond at the bottom represents the pooled effect size from a random-effects model, with width corresponding to the 95% CI. Heterogeneity statistics are shown ($Q = 271.76$, $df = 4$, $p = 1.33e-57$; $I^2 = 98.5\%$).

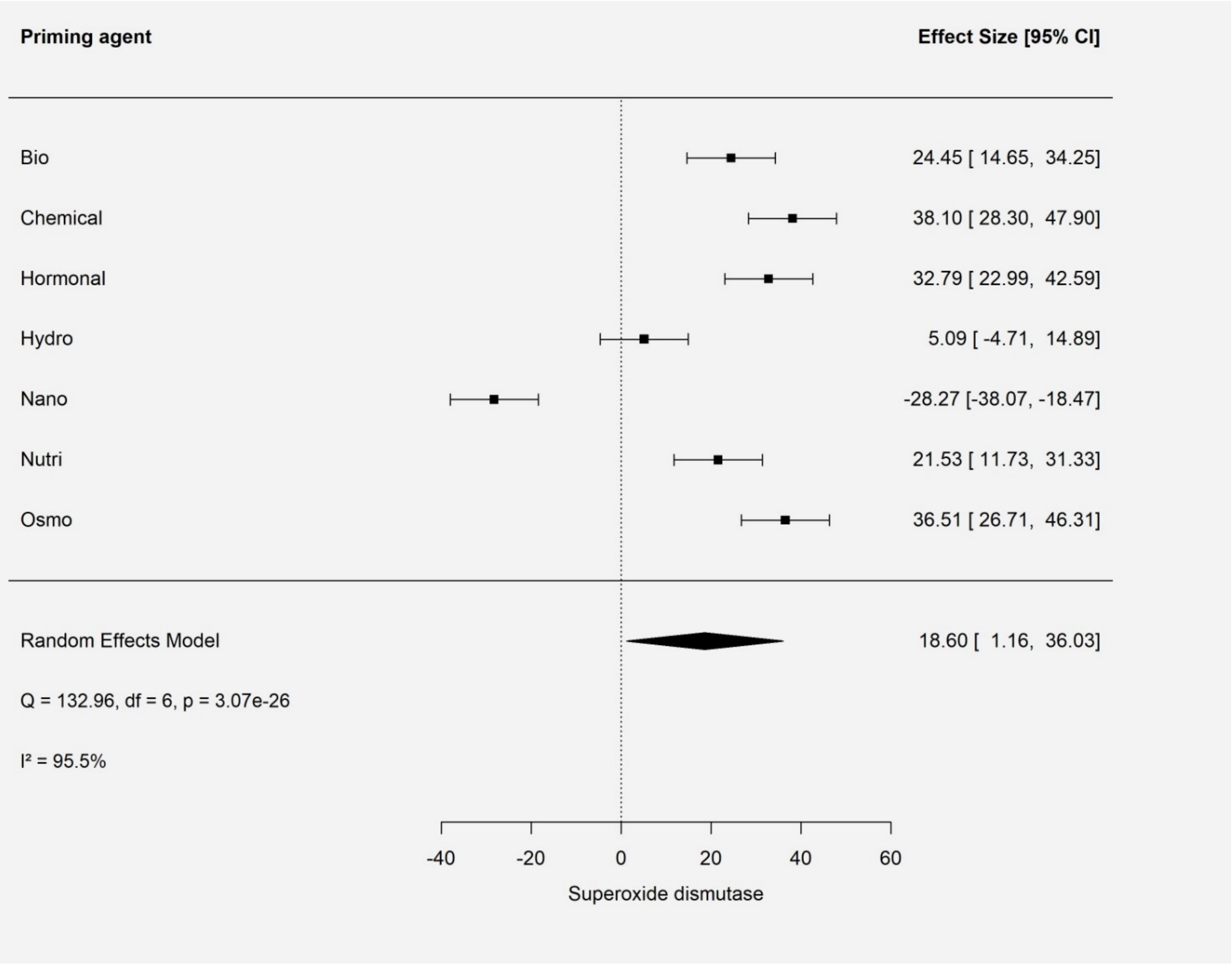


**Supplementary Figure S9:** Effect of different priming agents on superoxide dismutase. Plot showing the effect sizes (mean differences) and 95% confidence intervals (CI) for superoxide dismutase following treatment with priming agents: Bio, Chemical, Hormonal, Hydro, Nano, Nutri, and Osmo. Negative values indicate a reduction in superoxide dismutase relative to control, while positive values indicate an increase. Each black square represents the effect size for an individual priming agent, with horizontal lines representing the 95% CI. The diamond at the bottom represents the pooled effect size from a random-effects model, with width corresponding to the 95% CI. Heterogeneity statistics are shown ($Q = 132.96$, $df = 6$, $p = 3.07e-26$; $I^2 = 95.5\%$).

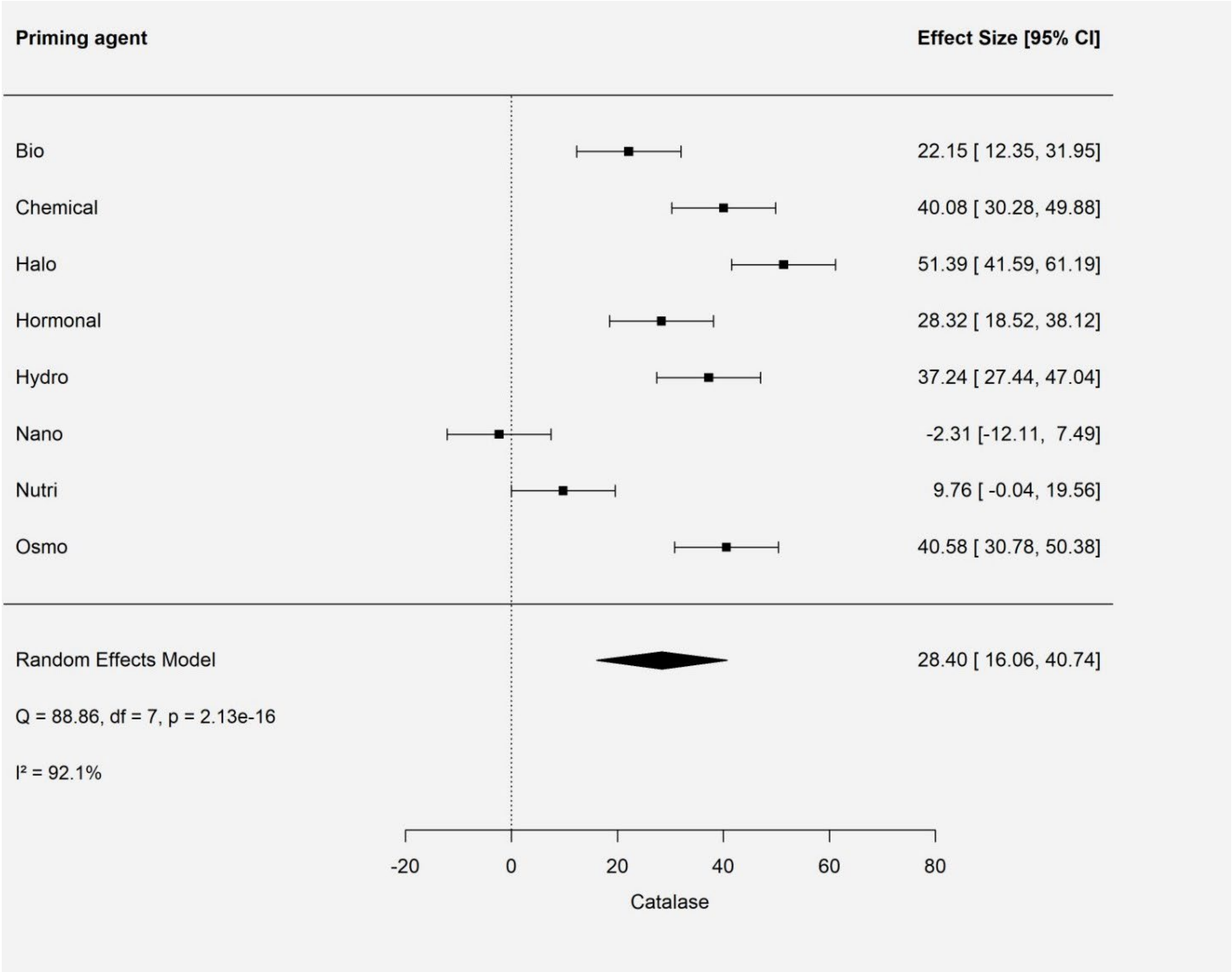


**Supplementary Figure S10:** Effect of different priming agents on catalase. Plot showing the effect sizes (mean differences) and 95% confidence intervals (CI) for catalase following treatment with priming agents: Bio, Chemical, Halo, Hormonal, Hydro, Nano, Nutri, and Osmo. Negative values indicate a reduction in catalase relative to control, while positive values indicate an increase. Each black square represents the effect size for an individual priming agent, with horizontal lines representing the 95% CI. The diamond at the bottom represents the pooled effect size from a random-effects model, with width corresponding to the 95% CI. Heterogeneity statistics are shown ($Q = 88.96$, $df = 7$, $p = 2.13e\text{-}16$; $I^2 = 92.1\%$).

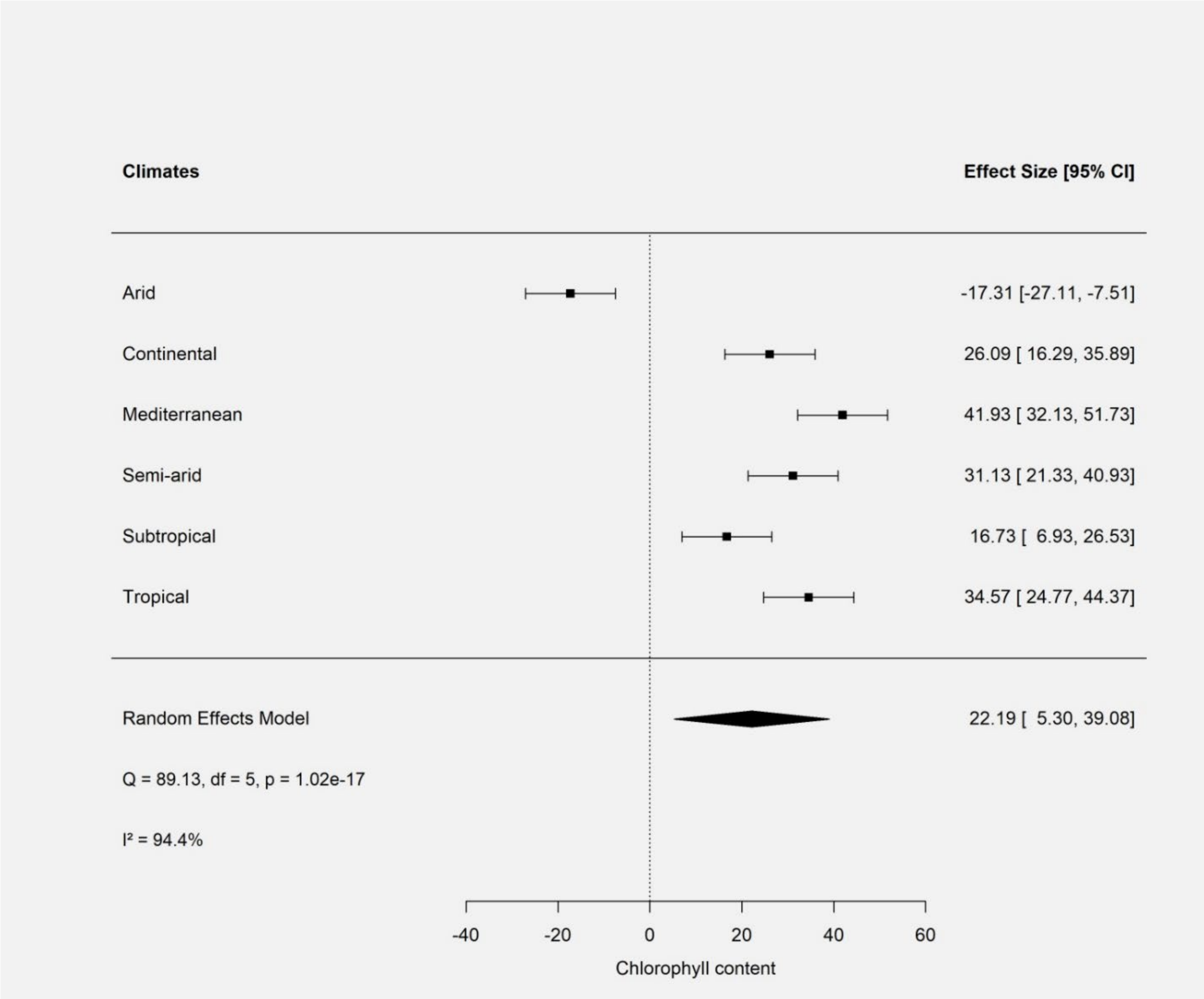


**Supplementary Figure S11:** Effect of climatic conditions on chlorophyll content. Plot showing the effect sizes (mean differences) and 95% confidence intervals (CI) for chlorophyll content following climatic conditions: Arid, Continental, Mediterranean, Semi-arid, Subtropical and Tropical. Negative values indicate a reduction in chlorophyll content relative to control, while positive values indicate an increase. Each black square represents the effect size for an individual climatic condition, with horizontal lines representing the 95% CI. The diamond at the bottom represents the pooled effect size from a random-effects model, with width corresponding to the 95% CI. Heterogeneity statistics are shown ($Q = 89.13$, $df = 5$, $p = 1.02e-17$; $I^2 = 94.4\%$).

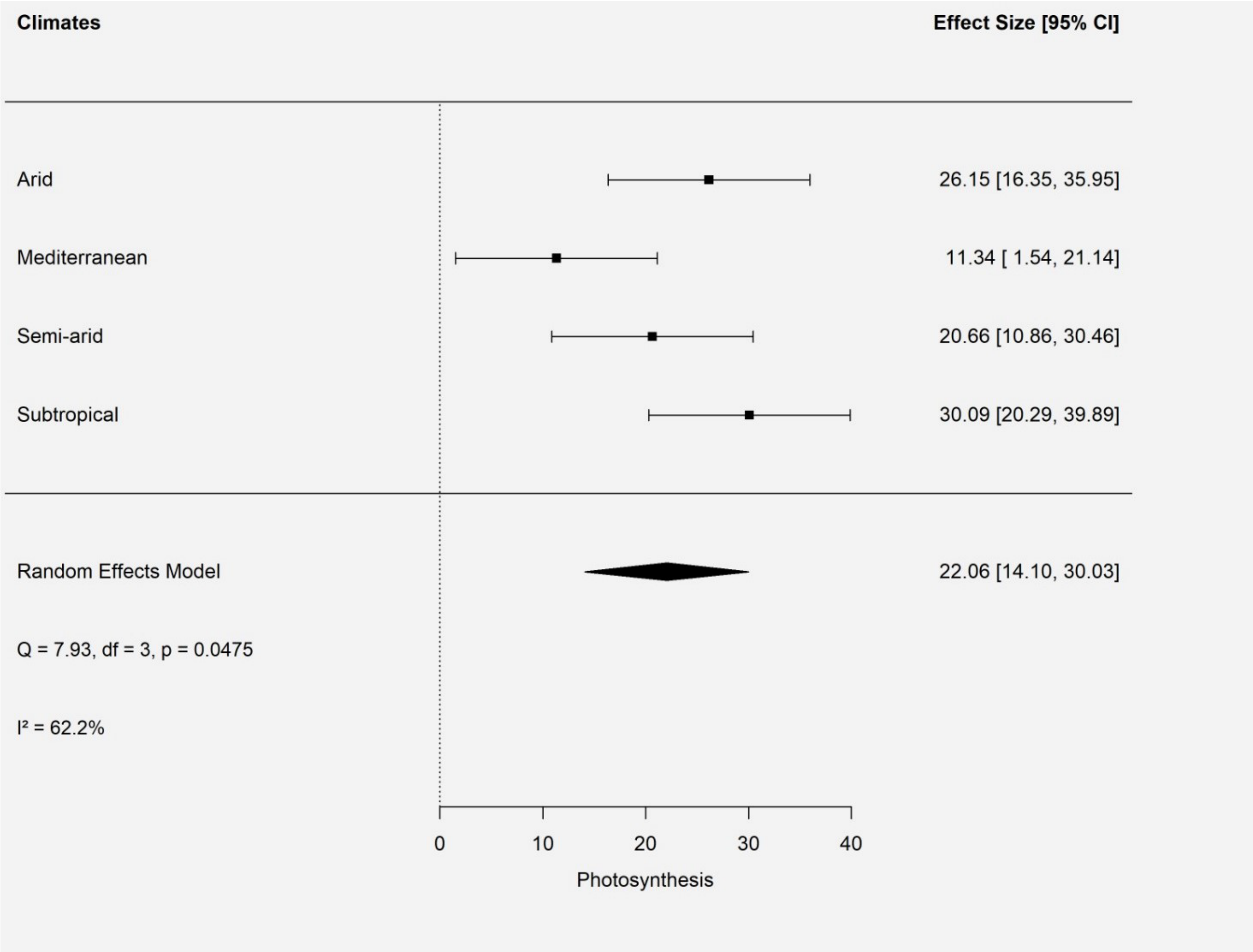


**Supplementary Figure S12:** Effect of climatic conditions on photosynthesis. Plot showing the effect sizes (mean differences) and 95% confidence intervals (CI) for photosynthesis following climatic conditions: Arid, Mediterranean, Semi-arid, and Subtropical. Negative values indicate a reduction in photosynthesis relative to control, while positive values indicate an increase. Each black square represents the effect size for an individual climatic condition, with horizontal lines representing the 95% CI. The diamond at the bottom represents the pooled effect size from a random-effects model, with width corresponding to the 95% CI. Heterogeneity statistics are shown ($Q = 7.93$, $df = 3$, $p = 0.0475$; $I^2 = 92.2\%$).

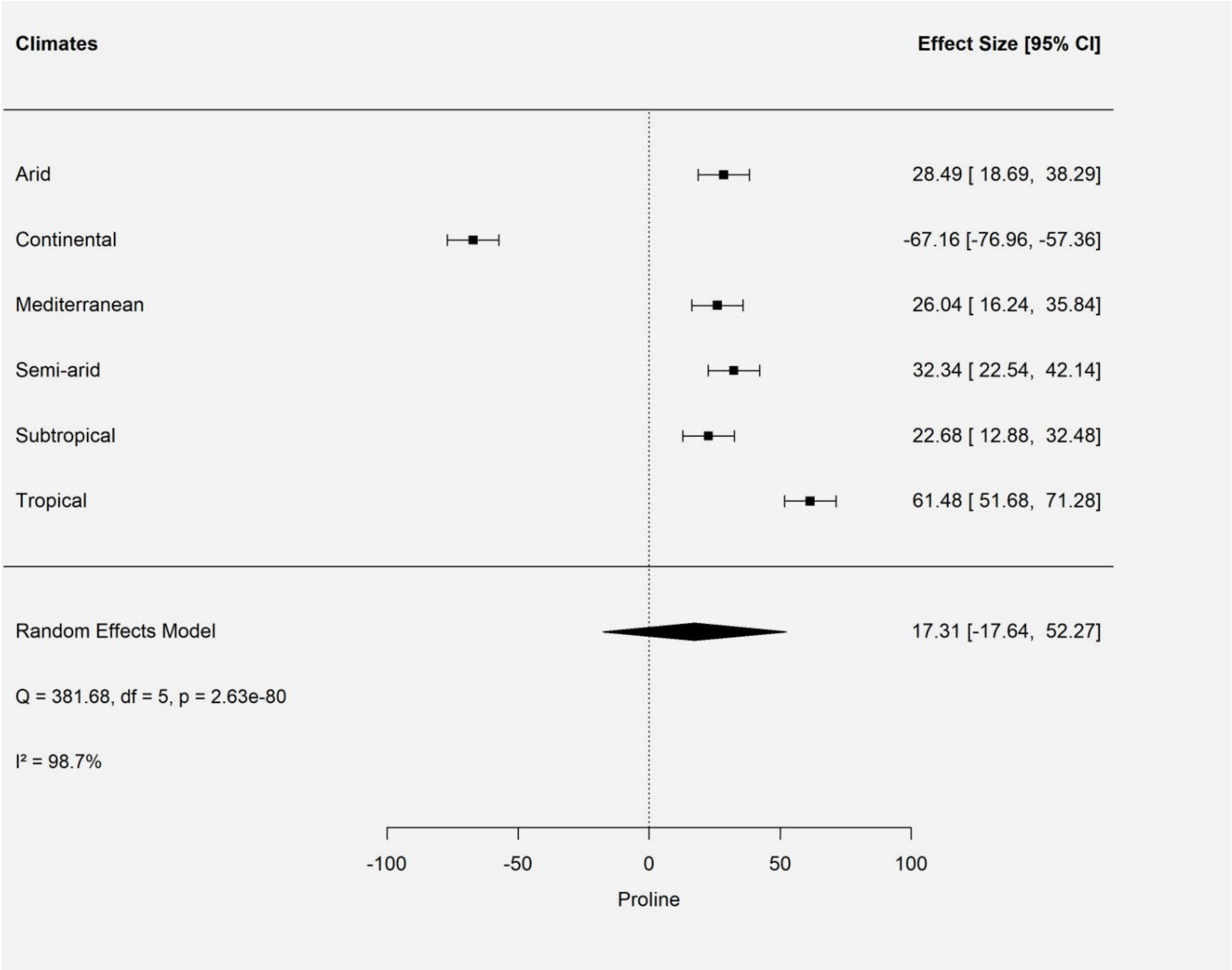


**Supplementary Figure S13:** Effect of climatic conditions on proline content. Plot showing the effect sizes (mean differences) and 95% confidence intervals (CI) for proline following climatic conditions: Arid, Continental, Mediterranean, Semi-arid, Subtropical and Tropical. Negative values indicate a reduction in proline relative to control, while positive values indicate an increase. Each black square represents the effect size for an individual climatic condition, with horizontal lines representing the 95% CI. The diamond at the bottom represents the pooled effect size from a random-effects model, with width corresponding to the 95% CI. Heterogeneity statistics are shown ($Q = 381.68$, $df = 5$, $p = 2.63e-80$; $I^2 = 98.7\%$).

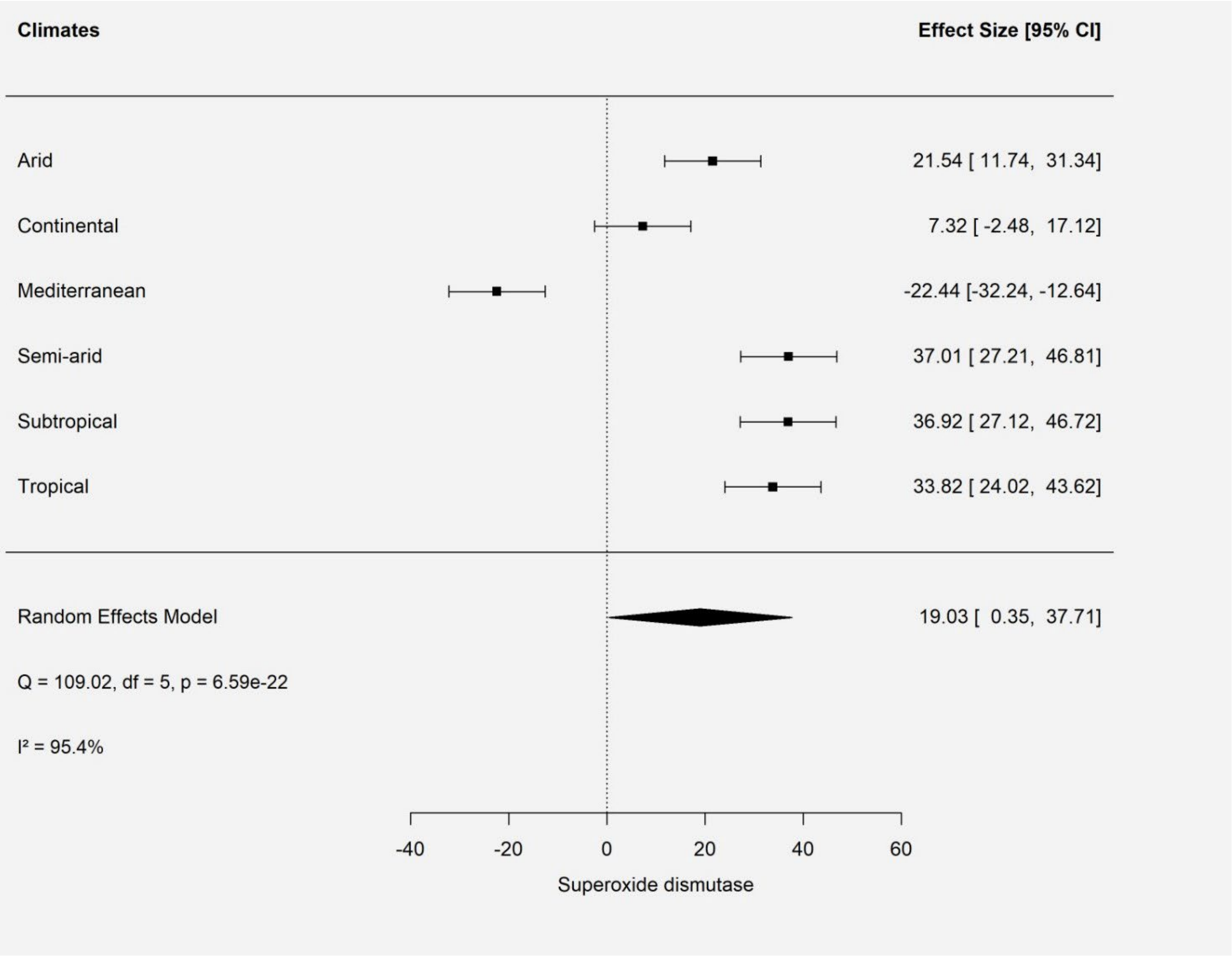


**Supplementary Figure S14:** Effect of climatic conditions on superoxide dismutase. Plot showing the effect sizes (mean differences) and 95% confidence intervals (CI) for superoxide dismutase following climatic conditions: Arid, Continental, Mediterranean, Semi-arid, Subtropical and Tropical. Negative values indicate a reduction in superoxide dismutase relative to control, while positive values indicate an increase. Each black square represents the effect size for an individual climatic condition, with horizontal lines representing the 95% CI. The diamond at the bottom represents the pooled effect size from a random-effects model, with width corresponding to the 95% CI. Heterogeneity statistics are shown ($Q = 109.02$, $df = 5$, $p = 6.59e-22$; $I^2 = 95.4\%$).

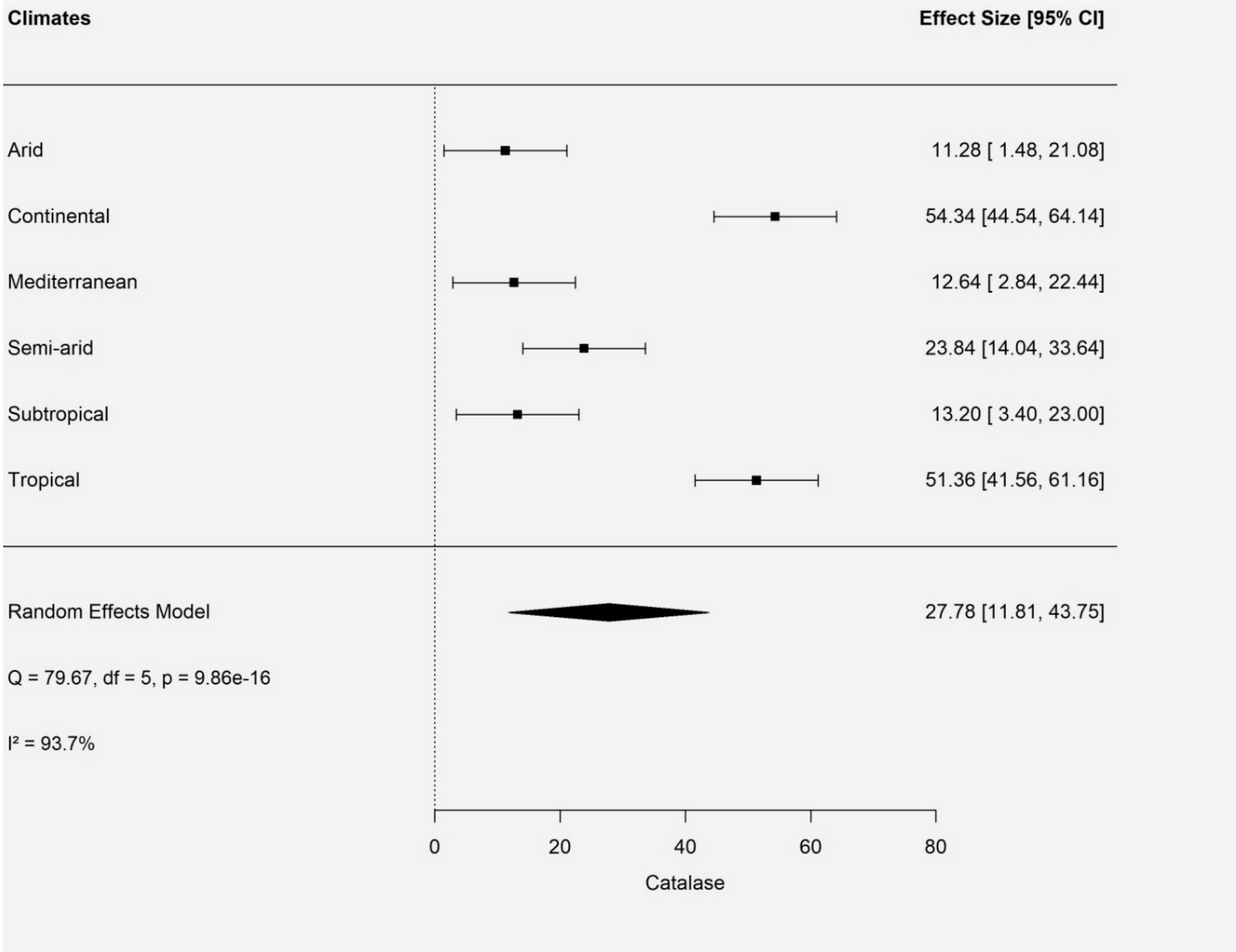


**Supplementary Figure S15:** Effect of climatic conditions on catalase. Plot showing the effect sizes (mean differences) and 95% confidence intervals (CI) for catalase following climatic conditions: Arid, Continental, Mediterranean, Semi-arid, Subtropical and Tropical. Negative values indicate a reduction in catalase relative to control, while positive values indicate an increase. Each black square represents the effect size for an individual climatic condition, with horizontal lines representing the 95% CI. The diamond at the bottom represents the pooled effect size from a random-effects model, with width corresponding to the 95% CI. Heterogeneity statistics are shown ($Q = 79.67$, $df = 5$, $p = 9.86\text{-}16$; $I^2 = 93.7\%$).
